# Influence of dominant sequence artifacts on generative AI for virtual contrast-enhancement in breast MRI

**DOI:** 10.1186/s12880-026-02996-8

**Published:** 2026-11-03

**Authors:** Luise Brock, Ameya Markale, Tri-Thien Nguyen, Marie Metz, Aju George, Shirin Heidarikahkesh, Hannes Schreiter, Julian Pfann Hossbach, Lorenz A. Kapsner, Sabine Ohlmeyer, Evelyn Wenkel, Michael Uder, Frederik B. Laun, Florian Knoll, Sebastian Bickelhaupt

**Affiliations:** 1 Institute of Radiology, Universitätsklinikum Erlangen, Friedrich-Alexander-Universität Erlangen-Nürnberg (FAU), Maximiliansplatz 3, 91054 Erlangen, Germany; 2 Department Artificial Intelligence in Biomedical Engineering, Friedrich-Alexander- Universität Erlangen-Nürnberg, Erlangen, Germany; 3 Institute of Computer Science, Polish Academy of Science, Warsaw, Poland; 4 Department for Computer Science 5 (Pattern Recognition), Friedrich-Alexander-University Erlangen-Nuremberg (FAU), Martensstrasse 3, 91058 Erlangen, Germany; 5 Medical Informatics, Friedrich-Alexander-Universität Erlangen-Nürnberg, Erlangen, Germany; 6 Diagnostische Radiologie, Radiologie München, München, Germany

**Keywords:** Magnetic resonance imaging, Deep learning, Artifacts, Diffusion weighted imaging, Breast cancer

## Abstract

**Background:**

Virtual contrast-enhanced (vCE) breast MRI is a deep learning approach for generating contrast-like images from non-contrast acquisitions. As diffusion-weighted imaging (DWI) has emerged as a major contributor to enhancement synthesis, understanding the influence of DWI-related image artifacts is important for the robustness and potential clinical implementation of AI-based contrast-free imaging approaches. We evaluated the robustness of a pix2pix-based vCE synthesis model to artifact-contaminated DWI during training and inference.

**Materials and methods:**

This IRB-approved, retrospective study included 1,974 multiparametric breast MRI examinations (mean age: 49 years) acquired with two clinical routine 3T MRI scanners (2017–2022). High b-value (b = 1500 s/mm^2^) DWI images were automatically classified for high-intensity (HI) and low-intensity (LI) artifacts using a pretrained neural network. A pix2pix 2D-GAN was trained either after excluding artifact-affected slices (vCE-clean) or using all available slices (vCE-all). Performance was evaluated using image similarity metrics, lesion-based analysis, reader assessment, and correlation of artifact scores with reconstruction errors.

**Results:**

Excluding artifact-affected slices during training resulted in a small but statistically significant increase in whole-image structural similarity index (SSIM) with small effect size (*vCE-all* 0.843 ± 0.034; *vCE-clean* 0.849 ± 0.031, *p* < 0.001, d_av_ = 0.20). However, lesion-based metrics and qualitative ratings of lesion enhancement and image quality were comparable between models. During Inference, strong LI DWI artifacts were associated with higher lesion-based errors.

**Conclusion:**

Controlling for artifacts on diffusion weighted imaging (DWI) during vCE training only minorly influenced technical metrics but not perceived image quality/lesion enhancement. In contrast, severe signal-loss artifacts during inference impaired local performance, highlighting the importance of image quality control during potential deployment.

**Supplementary Information:**

The online version contains supplementary material available at https://doi.org/10.1186/s12880-026-02996-8.

## Background

### Clinical role of contrast-enhanced breast MRI

Breast magnetic resonance imaging (MRI) is routinely performed using multiparametric acquisitions. The protocol commonly includes unenhanced T1-weighted, T2-weighted and diffusion-weighted images (DWI) as well as dynamic acquisitions acquired after the injection of gadolinium-based contrast agents (GBCA) [1, 2].

GBCA administration significantly supports the visualization of abnormal tissue structures in the breast tissue by enhancing areas with a disturbed extravascular leakage or an altered neoangiogenesis [3]. This information is crucial for both identifying and characterizing potentially suspicious areas in the breast, warranting further clarification [4].

GBCA-enhanced breast MRI is the method of choice for various clinical indications, such as clarification of suspected multifocal disease, relapse, or neoadjuvant chemotherapy response assessments [3, 5]. Therein, GBCA-enhanced breast MRI is the modality reported with the highest sensitivity for the detection of invasive breast cancer [6].

The pivotal diagnostic capability has raised interest in using GBCA-enhanced breast MRI in breast cancer screening. This is of particular interest for women with dense or very dense breast tissue on x-ray mammography, which is the routine modality for breast cancer screening, as mammographic sensitivity in this group can be substantially reduced [7], leaving a considerable proportion of malignancies undetected. Several studies have shown that these women benefit from increased cancer detection rates when breast MRI is offered as a supplemental imaging modality, as established in the DENSE trial and reported in interim results of the ongoing BRAID trial [8–10]. Consequently, recent recommendations, including those of the European Society of Breast Imaging (EUSOBI), advise supplemental breast MRI for women with very dense breast tissue [11].

### Motivation for contrast-free imaging approaches

Using GBCA outside of the clinical setting and within a preventive screening scenario, which includes healthy women, poses additional challenges. GBCAs are considered very safe and diagnostically important medications in MR imaging; however, rare side effects can occur, including reported cases of gadolinium deposition [12, 13]. The application and the necessity for continuous supervision by medical doctors causes additive constraints with regard to the applicability and accessibility, limiting its widespread use for screening purposes despite its unchallenged diagnostic capabilities [14].

To address these limitations, substantial research efforts have focused on developing breast MRI screening approaches that reduce or eliminate the need for GBCA administration [15, 16]. In this context, artificial intelligence-based methods have been increasingly explored to compensate for some of the limitations of unenhanced breast MRI. In particular, virtual contrast enhancement (vCE) aims to synthesize contrast-enhanced-like images from native, non-contrast MRI sequences using neural networks trained to learn characteristic enhancement patterns of breast tissue and lesions [17–20].

### Related work in virtual dynamic contrast enhancement (vDCE/vCE)

Several studies have demonstrated the feasibility of using deep learning to generate synthetic contrast-enhanced breast MRI from native acquisitions. An early study by Wang et al. showed that the first post-contrast dynamic phase could be synthesized from non-contrast MRI scans, thereby supporting lesion identification [21]. Subsequently, Chung et al. employed a 3D fully convolutional neural network incorporating multiparametric inputs, such as T1-weighted, T2-weighted, DWI, and Apparent Diffusion Coefficient (ADC) images. They demonstrated a high degree of similarity between synthetic and real contrast-enhanced images across 540 examinations [22].

More recently, George et al. compared pix2pix GAN and U-Net architectures for vCE generation from unenhanced T1-weighted, T2-weighted, and DWI images. While the U-Net architecture demonstrated superior quantitative performance, both approaches yielded visually realistic synthetic images in a reader study [17].

Furthermore, Liebert et al. systematically investigated the contribution of different native input sequences, demonstrating that models incorporating multi-b-value DWI significantly outperformed those without DWI in terms of quantitative metrics and qualitative lesion depiction. This suggests that DWI may be a dominant contributor to enhancement synthesis [19].

Together, these studies demonstrate the feasibility of vCE breast MRI and emphasize the importance of selecting appropriate input sequences. However, these studies were conducted using datasets that largely assumed diagnostically acceptable image quality and focused primarily on improving synthesis performance under ideal acquisition conditions. Consequently, it remains unclear how robust current vCE models are when confronted with clinically relevant image degradation, particularly artifacts affecting DWI, which has been identified as one of the most influential input sequences for enhancement synthesis. This represents an important barrier to the clinical translation of vCE techniques.

### Importance of input image quality and artifacts

The clinical reliability of vCE critically depends on the quality and consistency of the input images used for both training and inference and may be particularly vulnerable when the dominant input sequence is degraded [19]. To date, the impact of input image artifacts on vCE performance has received little attention, with previous studies generally assuming diagnostically acceptable input image quality rather than explicitly evaluating model robustness to image degradation. Generative models are known to be sensitive to artifacts, spatial inconsistencies, and intensity variations, which may be transferred into the synthesized images and result in e.g., erroneous enhancement patterns [23, 24].

In breast MRI, this concern is particularly relevant for DWI, given its susceptibility to signal intensity-related artifacts. Such artifacts can be caused amongst by insufficient fat saturation or surface coil flare artifacts, causing high signal intensity areas, or by signal voids causing black-out areas within the tissue [25–27]. While high-intensity artifacts introduce spurious signal that may mimic tissue contrast, low-intensity artifacts result in localized signal loss and may obscure anatomical structures and lesion-related diffusion information. Because DWI may provide the strongest contribution to enhancement simulation, artifacts in this sequence could disproportionately affect synthesized vCE images and, in particular, lesion conspicuity [19]. During model training, corrupted inputs may bias the learned mapping between native and contrast-enhanced appearances of focal lesions, whereas during inference they may propagate into the generated images and alter lesion visibility by mimicking or obscuring lesion enhancement.

### Study objective

Therefore, to address the lack of evidence regarding the robustness of virtual contrast enhancement to degraded input image quality, our study investigated the influence of DWI image quality differentiated into high-intensity (HI) artifacts and low-intensity (LI) artifacts on both training and inference performance of a neural network for the creation of vCE breast MRI images. Specifically, we compared artifact-aware versus non-selective training strategies and assessed whether artifact severity during inference affects lesion depiction, and visual image quality. By addressing the impact of artifact-related image degradation, this study aims to advance the understanding of technical factors influencing the robustness of AI-based contrast-free breast MRI.

## Materials and methods

### Summary of patient cohort and MRI protocol

This retrospective IRB-approved study included *n* = 1,974 routine breast MRI acquisitions performed between January 2017 and June 2022 at the University Hospital Erlangen using two 3 Tesla MRI systems (Magnetom Skyra Fit and Magnetom Vida, Siemens Healthineers, Erlangen, Germany) and an 18-channel breast coil (Siemens Healthineers, Erlangen, Germany). All routine breast MRI examinations performed during the study period that fulfilled the inclusion criteria were eligible for analysis. Due to the retrospective character of the study the requirement for written informed consent was waived. Data were accessed for research purposes in batches between April 2021 and October 2024. Only the principal investigator had the possibility to access information that could identify individual participants during or after data collection. Examinations of patients with breast implants were excluded. The dataset included into the study has previously been partially included in research on breast MRI, however investigating the influence of artifacts on virtual contrast enhancement generation has not been part of the previous experiments.

The examinations included a routine multiparametric breast MRI protocol consisting of T2-weighted (T2w), T1-weighted (T1w), T1-weighted dynamic contrast-enhanced (DCE) imaging, the corresponding subtraction images, and DWI sequences acquired with a set of three b-values (b = 50, 750, and 1,500 s/mm^2^). The T1w sequence was acquired with a slice thickness of 1.5–1.8 mm (repetition time (TR) 5.51–5.97 ms, echo time (TE) 2.46 ms), the T2w sequence with a slice thickness of 4 mm (TR 3570–5670 ms, TE 60–70 ms), and the DWI sequence with a slice thickness of 4 mm (TR 5810–13440 ms, TE 66–80 ms). Detailed acquisition parameters are summarized in Supplemental Table 1.

The dataset was divided into training (*n* = 1,521), validation (*n* = 237), and test (*n* = 216) sets. Differences in demographic and acquisition characteristics between the dataset subsets were assessed statistically. Age was compared using one-way analysis of variance (ANOVA), followed by Tukey’s honestly significant difference test for post-hoc pairwise comparisons. BMI was compared using the Kruskal–Wallis test, followed by Dunn’s test with Holm correction for multiple pairwise comparisons. Scanner distributions were compared using the chi-square test of independence. Effect sizes were quantified using eta-squared (η²), epsilon-squared (ε²), and Cramér’s V, respectively. A two-sided p-value < 0.05 was considered statistically significant. Preprocessing followed the approach described by Liebert et al. [19] and comprised field-of-view (FoV) resampling, alignment along the z-dimension, z-score intensity normalization, intensity clamping, and rescaling. After preprocessing, the images had an in-plane resolution of (280 × 448) with an average of 118 slices per subject.

### Study Setup

Our goal was to examine the impact of artifacts presenting as high-intensity areas (HI) or as areas of signal-void/loss (Low-Intensity, LI) on the high b-value DWI input sequence on a generative AI model (vCE network) for synthesizing contrast-enhanced breast MRI.

First, artifact-affected slices were identified for both HI and LI artifacts using the multiclass artifact classification framework described below in (“DWI image quality and artifact assessment”), generating a CSV file containing slice-wise artifact scores for each case.

The vCE network architecture followed the implementation by George et al. [17], and two training strategies were compared:

1. training on the full dataset without artifact-based exclusion, called *vCE-all*, and.
2. training with exclusion of slices with severe artifacts (score = 4) across all involved sequences, called *vCE-clean*.

**Fig. 1:**
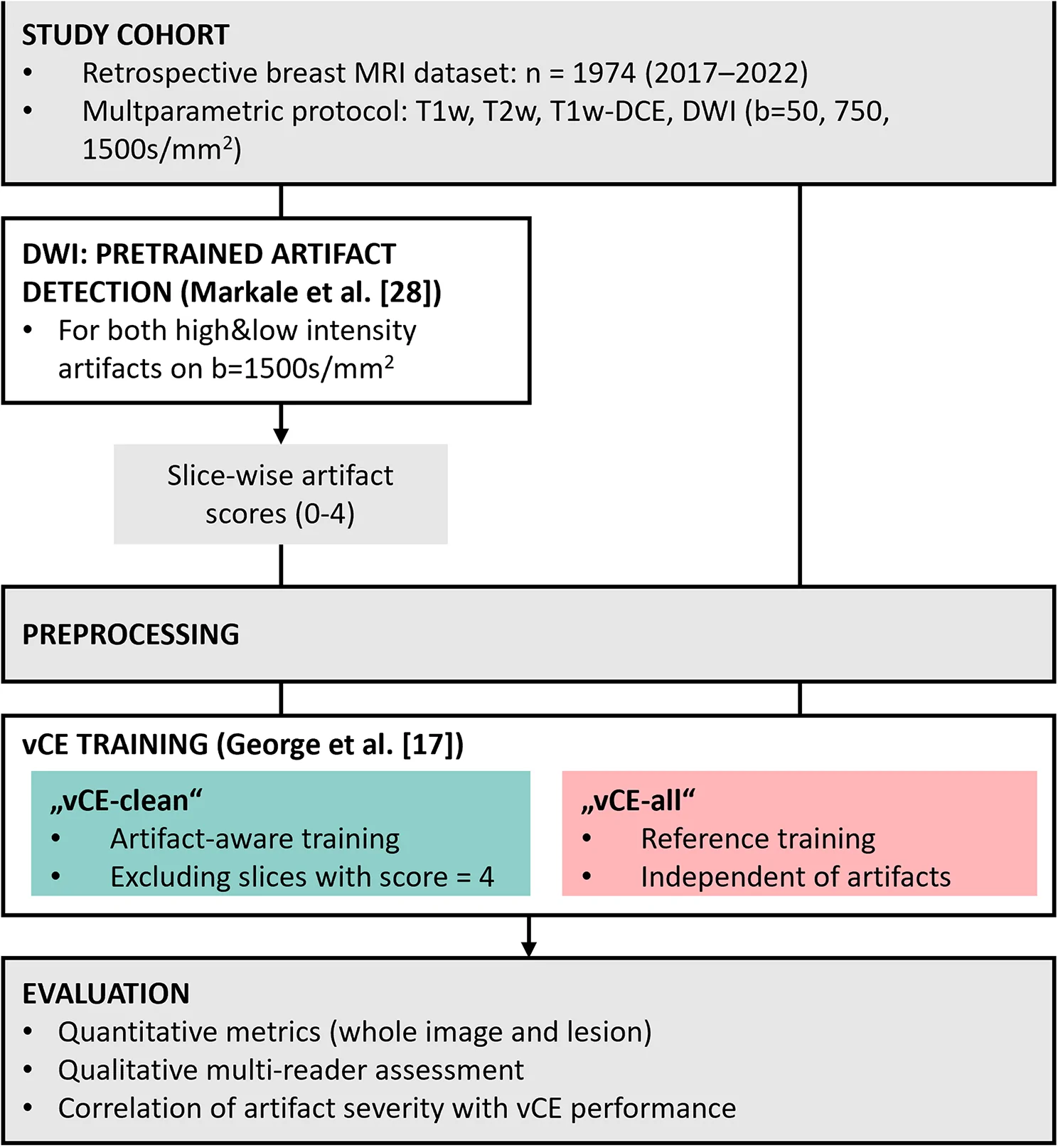
Overview of the study setup. Breast MRI examinations (n = 1,974) acquired between 2017 and mid-2022 underwent preprocessing. A pretrained artifact detection network provided slice-wise artifact scores (by Markale et al. [28, 29]). Based on these scores, two virtual contrast enhancement (vCE) training strategies were compared: training on the full dataset (vCE-all) and artifact-aware training with exclusion of slices with severe intensity related artifacts (score = 4) across all MRI sequences (vCE-clean). vCE model was based on George et al. [17]. Model performance was evaluated using quantitative similarity metrics, qualitative image assessment, and correlation analyses with artifact severity

Model performance was evaluated using quantitative similarity metrics computed for both whole images and lesion regions, complemented by a qualitative assessment of image quality and lesion enhancement. Additionally, correlations between artifact scores and quantitative performance metrics were analyzed in the test set, where no slice exclusion was applied during inference. The study setup is also summarized in Fig. 1.

### DWI image quality and artifact assessment

To ensure slice number consistency for artifact detection and vCE training, the DWI data were first resampled to match the size and spatial resolution of the T1-weighted images. No additional preprocessing was applied prior to artifact scoring, as the pretrained artifact detection networks from Markale et al. inherently did further pre-processing for network training, such as normalization. All remaining preprocessing steps described in Sect.  2.2 were applied only after artifact scoring.

The dataset that was originally used to train the classification networks (151 examinations) is fully included in our study cohort. However, this overlap does not bias the present analysis because the artifact detection model was neither retrained nor evaluated here; it was used exclusively as a fixed, pretrained model to provide artifact labels and investigate the effect of artifact removal on vCE network training.

Separate multiclass classification networks using the DenseNet121 architecture were used to detect HI and LI DWI artifacts. Each network was pretrained by Markale et al. and produced a slice-wise and breast-side-wise artifact score ranging from 0 (no artifact) to 4 (severe artifact), resulting in two CSV files per case [28, 29]. To derive a single artifact label for each slice in the downstream training process, the network output was modified to provide one HI and one LI score for the entire slice, selecting the higher score from both breast sides. Then, HI and LI scores were merged by also selecting the higher of the two values. This ensured that strong artifacts of either category were reflected in the combined artifact representation.

### vCE neural network architecture and training

**Fig. 2:**
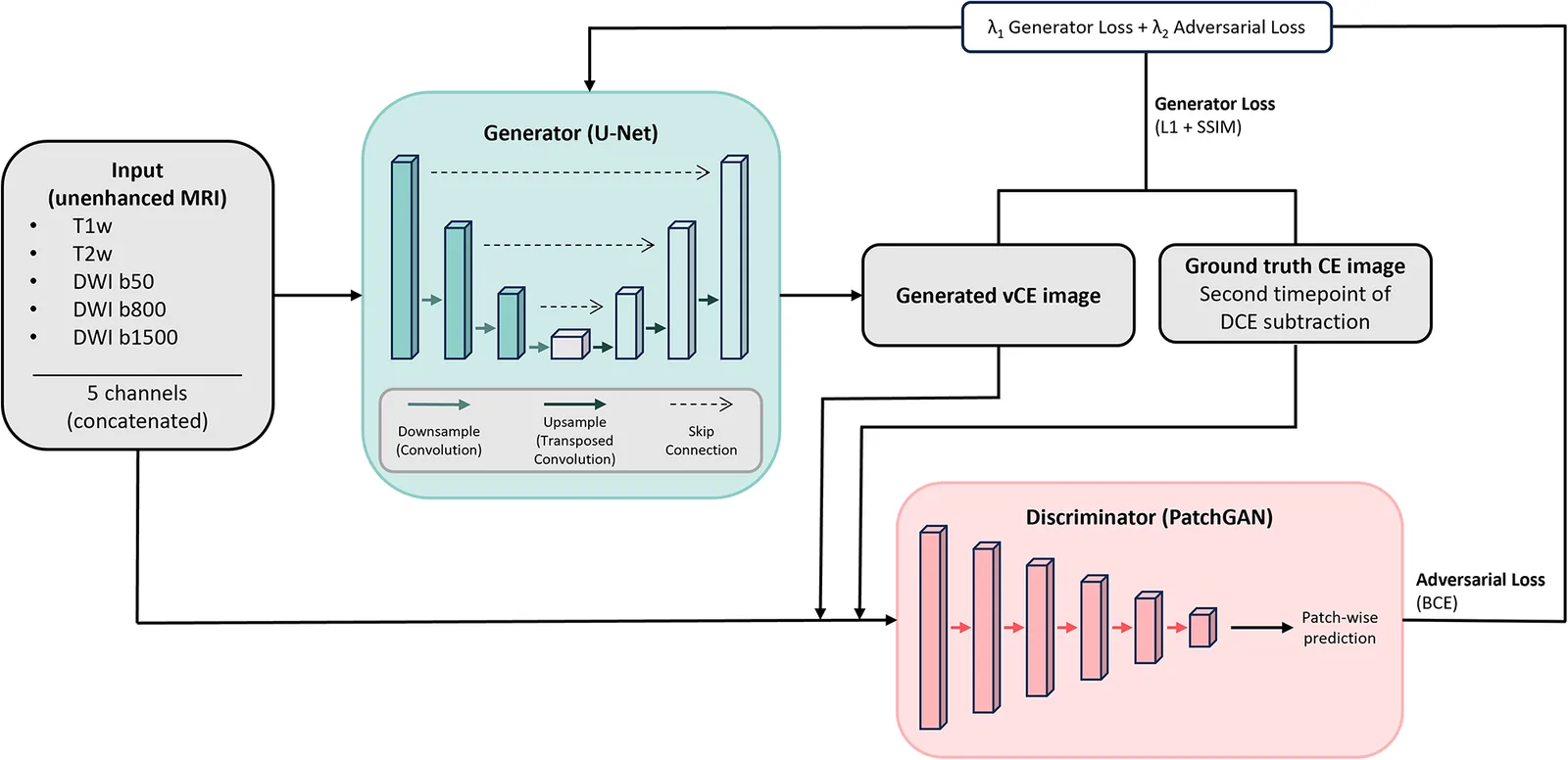
Schematic overview of the pix2pix GAN architecture used for virtual contrast-enhanced (vCE) image synthesis. Five-channel multiparametric breast MRI (T1w, T2w, and three DWI images) is provided as input to a U-Net-based generator, which synthesizes a single-channel virtual contrast-enhanced image. During training, the generated vCE image or the corresponding ground-truth contrast-enhanced image is concatenated with the multiparametric MRI input and presented to a PatchGAN discriminator, which performs patch-wise real/fake discrimination. The generator is optimized using a combination of generator losses (L1 and SSIM) and an adversarial loss. During inference, only the trained generator is used to generate vCE images

For generating vCE breast MRI images, we employed the GAN architecture proposed by George et al., following the preprocessing pipeline described by Liebert et al. [17, 19]. The network consists of a U-Net-based generator that synthesizes the second timepoint of the T1w DCE subtraction images from the multiparametric MRI input and a PatchGAN discriminator that distinguishes synthetic from real contrast-enhanced images, thereby encouraging the generation of visually realistic image characteristics through adversarial training (Fig. 2). The network was trained for 65 epochs using all available sequences as input (T2w, T1w, and the three DWI images), with the target being the second timepoint of the DCE subtraction sequence. Two separate training strategies were applied: *vCE-all*, which included all slices from the training and validation sets, and *vCE-clean*, which excluded slices with a maximum HI or LI artifact score of 4, as determined by the pretrained artifact detection networks. Both training strategies used the identical patient-level training, validation, and test split, thereby ensuring that no patient was present in more than one subset. While multiple examinations per patient were present in the training split, there was only one examination per patient for validation and testing. The data splits were the same for both network trainings as described in Sect. 2.1 (training: *n* = 1,521, validation: *n* = 237, test: *n* = 216).

Training was performed with a batch size of 48, using learning rates of 1 × 10^− 4^ for the generator and 1 × 10^− 3^ for the discriminator. The optimizer type, loss functions, data augmentation, and learning rate scheduling were applied according to George et al. Specifically, identical data augmentation was applied for both training strategies and consisted of random horizontal and vertical flipping, each with a probability of 0.5. Hyperparameters were adopted from the previously published implementation by George et al. and were not further optimized for the present study. Model selection was based on performance on the validation set, optimizing both the overall structural similarity index measure (SSIM) of the generated images and the mean network lesion-region squared error (MSE) within lesion regions. To select the final model, the six epochs with the lowest lesion-region MSE were first identified. Among these candidate epochs, the epoch with the highest validation SSIM was selected for inference. For lesion region segmentations, we employed the nnU-Net model on the second timepoint of the DCE subtraction sequence as described in the MAMA-MIA study, which segmented enhancing areas in the breast MRI [30]. These automatically generated segmentations were used exclusively for lesion-focused model selection during training and were not manually reviewed for the present study.

The best-performing epochs were then used to run inference on the whole test set without slice exclusion for artifact affected slices.

### Evaluation

The performance of *vCE-all* and *vCE-clean* was evaluated using a combination of quantitative image quality and lesion metrics, qualitative expert reading, and correlation analysis, investigating potential correlations of artifact strength and quantitative metrics.

#### Quantitative analysis

Overall image quality was assessed by computing SSIM and whole-image MSE between the generated vCE images and the corresponding ground truth DCE subtraction images [31]. The normality of metric distributions was evaluated using the Shapiro–Wilk test [32]. For normally distributed data, a paired t-test was applied, and otherwise the Wilcoxon signed-rank test was used [33, 34]. For the primary paired quantitative comparisons, 95% confidence intervals (CI) of the mean paired differences were estimated using bias-corrected and accelerated (BCa) bootstrap resampling with 10,000 iterations, and effect sizes were quantified using Cohen’s d_av_, calculated as the mean paired difference divided by the average standard deviation of the two conditions [35, 36]. Statistical significance was defined as *p* < 0.05.

An essential objective of vCE is to reproduce lesion enhancement comparable to true contrast-enhanced imaging. Therefore, lesion behavior was additionally analyzed. For those cases in the independent test set containing mass lesions (Breast Imaging-Reporting and Data System (BI-RADs) 4–6), segmentations were available, resulting in 61 segmented lesions. These segmentations had been created previously by board-certified breast radiologists or trained medical students under expert supervision and were visually inspected before quantitative analysis to ensure adequate segmentation quality. The focus was not on the direct computation of SSIM and MSE within the lesion masks, since small spatial misalignments between generated images and the ground truth, caused by input misregistration, may lead to biased lesion-specific metrics. Nevertheless, a post-hoc normalized lesion MSE and SSIM were calculated and are reported in the Supplementary Material. For this analysis, the exported ground-truth and generated volumes were independently clipped to their respective 1st–99th percentile intensity ranges, scaled to ([0,1]), and evaluated within the lesion mask. This metric is distinct from the network lesion-region MSE used during model validation. Instead of focusing on lesion SSIM and MSE, lesion appearance was evaluated within a padded three-dimensional bounding box surrounding each lesion (lesion bounding box plus 5 voxels in all directions). Within this region, the Kullback-Leibler (KL) divergence between the ground truth and vCE intensity histograms was computed, and lesion peak intensity characteristics were compared by identifying the peak of the ground truth histogram and measuring the corresponding peak height in the vCE histogram [37]. This approach allowed assessment of whether lesions exhibited comparable enhancement magnitude and relative size, independent of exact spatial alignment.

#### Qualitative analysis

Qualitative evaluation was performed by three independent readers (R1: M.M., 0.5 years of breast MRI experience; R2: T.T.N., board-certified radiologist with 5 years of experience; and R3: S.B., board-certified radiologist with over 10 years of experience in breast MRI). Two separate reading studies were conducted.

For the lesion enhancement reading, the central slice of the target lesions (in case of multiple lesions defined as the largest lesion) was selected for each of the 61 breast MRI examinations from individual patients in the independent test set with lesions, and a bounding box including the lesion with an additional 5-pixel margin was extracted. For each case, readers were presented with three corresponding images: the ground truth contrast enhanced subtraction image from the DCE sequence, the *vCE-clean* output, and the *vCE-all* output. Images were displayed using a standardized visualization preprocessing, including lower-percentile clamping and bounding-box display around the lesion. Readers were asked to score whether enhancement was absent in both vCE images (0), comparable between both (1), better represented in *vCE-clean* (2), or better represented in *vCE-all* (3).

A second reading assessed the robustness of the image quality of the vCE predictions on slices with severe artifacts in the high b-value DWI input data. All DWI slices in the test-set which had been labeled with a maximum artifact score were visually reviewed and categorized to confirm artifact severity. Only slices with confirmed severe DWI artifacts were included. From this dataset 100 slices with severe HI artifacts and 100 with severe LI artifacts were randomly selected. The 200 slices were gathered from 62 individual patients. For each case, readers were presented with the ground truth contrast enhanced subtraction image and the two vCE output images from the *vCE-clean* or *vCE-all* network, next to each other, clamping the lower percentile for better visualization. Readers assessed and compared the image quality of the *vCE-clean* and *vCE-all* outputs and scored, using a “Turing-test-like” assessment where they were asked to indicate if image quality was comparable (1) or superior in one of the two networks (if *vCE-clean* was superior, score 2 was given, else score 3). Inter-reader agreement was assessed using pairwise percent agreement, calculated as the proportion of cases for which two readers assigned identical ratings. In addition, for each pair of readers Cohen’s κ was calculated to account for agreement beyond chance.

#### Correlation analysis

To investigate the influence of DWI artifact strength on vCE performance, correlation analyses were performed between artifact measures and multiple image quality and lesion-based performance metrics. Whole-image metrics included SSIM and MSE, which were evaluated on a slice-wise basis by correlating the artifact score of each slice with the corresponding metric value in that slice. Lesion-based metrics included KL divergence, lesion peak-height relation, and post-hoc normalized lesion MSE, which were analyzed per lesion by correlating the percentage of slices with artifact score 4 (highest score), in the following called strong-artifact-affected slices, within each lesion volume with the respective whole lesion-level metric.

Because the distribution of artifact scores was highly imbalanced toward slices without artifacts (score 0), all correlation analyses were performed both including all slices and restricting the analysis to slices with artifact scores > 0. For lesion-based analyses, lesions with 0% strong-artifact-affected slices were excluded in the restricted analysis.

Spearman’s rank correlation coefficient (ρ) was used to assess monotonic associations between artifact measures and performance metrics due to the ordinal nature of the artifact scores and non-normal distributions of several metrics [38]. Because these correlation analyses were exploratory, no multiplicity correction was applied for multiple testing. Corresponding p-values were reported. However, given the large number of slice-wise observations and the presence of multiple slices per patient, statistical significance was interpreted cautiously and emphasis was placed on effect sizes.

To assess whether the relationship between artifact strength and performance differed between vCE network variants (*vCE-clean* vs. *vCE-all*), an ANCOVA model with interaction terms was additionally applied, using the performance metric as dependent variable, artifact measure as covariate, and network version as categorical factor [39]. A significant interaction term would indicate a differential sensitivity of the two network variants to increasing artifact prevalence.

#### Exploratory scanner-stratified analysis

To explore potential scanner-related effects, additional subgroup analyses investigating the different artifact prevalences and performance differences for the two MRI systems (MAGNETOM Vida (*n* = 1,540) vs. MAGNETOM Skyra Fit (*n* = 434)). The examination-level proportions of slices assigned to each HI and LI artifact class were compared between scanners using Mann–Whitney U tests with Holm correction for multiple comparisons [40, 41]. In addition, whole-image and lesion-based SSIM and MSE were compared between *vCE-clean* and *vCE-all* within each scanner using paired Wilcoxon signed-rank tests. The whole-image metrics were analyzed on the hold-out test set (MAGNETOM Vida *n* = 123 and MAGNETOM Skyra Fit *n* = 93) and the lesion-based metrics were analyzed on the 61 cases with segmentations (MAGNETOM Vida *n* = 34 and MAGNETOM Skyra Fit *n* = 27). Scanner-specific differences were assessed using Mann–Whitney U tests. These analyses were considered exploratory.

## Results

### Demographics and clinical findings

The complete dataset represents a clinically diverse breast MRI population with a mean patient age of 49.4 ± 12.6 years and BI-RADS scoring available based on the clinical readings for each examination, see Table 1. All BI-RADS categories are present, and their overall distribution is consistent with routine clinical settings, with lower frequencies of intermediate categories. Age differed significantly across the three subsets (ANOVA, *p* < 0.001), with a small effect size (η² = 0.011); post-hoc analysis showed that the independent test set was significantly older than the training set (Tukey HSD, *p* < 0.001). BMI also differed across subsets (Kruskal–Wallis, *p* = 0.012), although the effect size was small (ε² = 0.003). Scanner distribution differed between the subsets (χ²(2) = 66.89, *p* < 0.001, Cramér’s V = 0.181). BI-RADS categories are dominated by scores 2 and 6 across all subsets, reflecting a clinically representative cohort.

**Table 1:** Overview of demographic characteristics for the training, validation, and independent test sets. Reported parameters include the number of patients and examinations, patient age and BMI, distribution of MAGNETOM Vida and Skyra Fit examinations, and BI-RADS classification. The BI-RADS score refers to the highest score given for an examination during routine clinical reading. BI-RADS categories reflect radiological assessment. Age and body mass index (BMI) is presented as the mean and standard deviation

Metric	Entire dataset	Training	Validation	Independent test set	
No. of patients	1482 (100.0%)	1029 (69.4%)	237 (16.0%)	216 (14.6%)	
No. of examinations	1974	1521	237	216	
Split ratio of examinations (%)	100	77.1	12	10.9	
Age (years)	49.4 ± 12.6	48.8 ± 12.3	50.2 ± 13.6	52.9 ± 12.6	
BMI (kg/m^2^)	25.2 ± 5.0	25.1 ± 4.9	25.1 ± 5.2	26.1 ± 5.1	
MAGNETOM Vida examinations	1540 (78.0%)	1238 (81.4%)	179 (75.5%)	123 (56.9%)	
MAGNETOM Skyra Fit examinations	434 (22.0%)	283 (18.6%)	58 (24.5%)	93 (43.1%)	
**Routine BI-RADS score**					
no score	142	140	2	0	
0	16	13	3	0	
1	23	18	3	2	
2	1053	829	109	115	
3	144	112	14	18	
4	180	132	27	21	
5	102	67	19	16	

### DWI image quality and artifact assessment

**Fig. 3:**
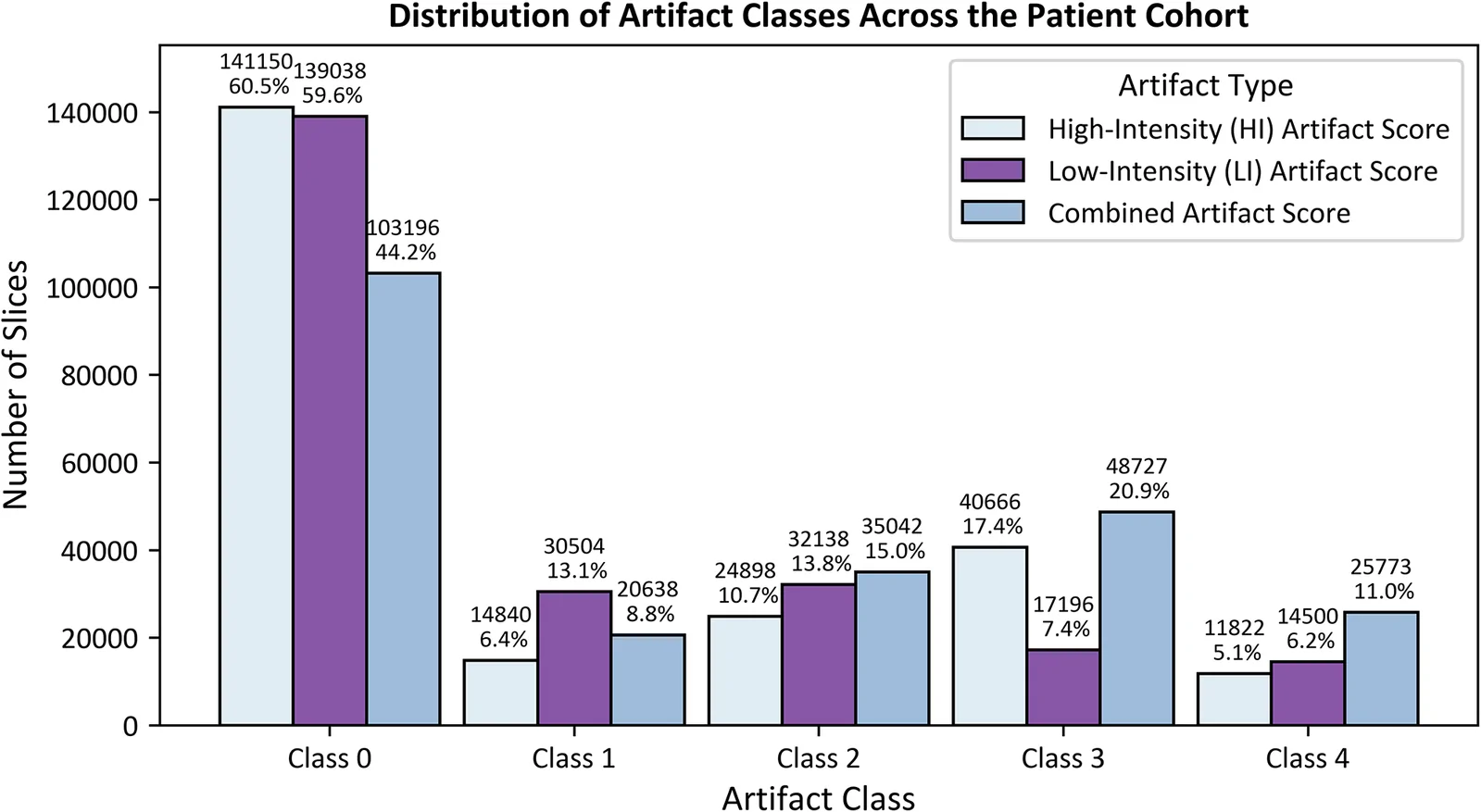
Distribution of DWI intensity artifact severity across slices. Artifact class distributions are shown for the high-intensity (HI) model, low-intensity (LI) model, and the combined artifact score obtained by selecting the higher of the two scores. The majority of slices were artifact-free (Class 0), while severe artifacts (Class 4) were relatively rare. Moderate artifact levels (Classes 2–3) constituted a relevant fraction of the data, indicating clinically meaningful artifact burden

DWI image quality was assessed using the pretrained DenseNet121-based artifact classification network proposed by Markale et al. [28]. The network was originally developed and validated on a subset of the breast MRI dataset used in the present study and achieved weighted accuracies of 0.59 and 0.61, AUROC values of 0.85 and 0.88, and AUPRC values of 0.75 and 0.69 for hyperintense and hypointense artifact classification [28]. The present study employed the network described in the original preprint [28], which slightly differs from the subsequently published journal version [29] in the ground-truth annotations used for training. Analysis of the high b-value DWI data using the pretrained HI and LI artifact detection network revealed that most DWI slices were found to be classified as artifact-free, see Fig. 3. Specifically, Class 0 slices accounted for 60.5% of the HI model, 59.6% of the LI model, and 44.2% of the combined HI + LI score. Severe artifacts (Class 4) were relatively uncommon, accounting for 5.1% of slices in the HI model, 6.2% in the LI model, and 11% in the combined HI + LI score. Moderate artifacts (Classes 2–3) were present in a significant proportion of the slices, making up about 36% of the combined (HI + LI) distribution. As expected, the combined HI + LI score shifted the distribution toward higher classes, reducing the proportion of slices without any artifacts. Analysis of the distributions across the training, validation, and test sets individually showed similar patterns, indicating that artifact prevalence was consistent across all data splits (see Supplemental Fig. 1).

### vCE Training and selection of training epoch for independent test set deployment

For the *vCE-clean* training, accordingly, 11% of the individual slices were excluded due to severe artifacts (HI or LI score 4). During training, SSIM increased in the early epochs and then stabilized with small fluctuations. Both networks showed similar behavior, with slightly higher SSIM values for vCE-clean. Based on whole-image SSIM and the network lesion-region MSE analysis on the validation set, epoch 60 of the training was selected for the *vCE-all network*, yielding a whole-image validation SSIM of 0.850 ± 0.042 and a lesion validation MSE of 0.127 × 10^− 3^ ± 0.247 × 10^− 3^. For *vCE-clean*, epoch 61 was selected, with a validation SSIM of 0.854 ± 0.041 and a lesion validation MSE of 0.131 × 10^− 3^ ± 0.256 × 10^− 3^. These results indicate that excluding slices with severe artifacts did not substantially alter the performance on the validation set. To further analyze the performance of the two networks on the test set, the best-performing epochs were used for inference.

#### Quantitative analysis

**Fig. 4:**
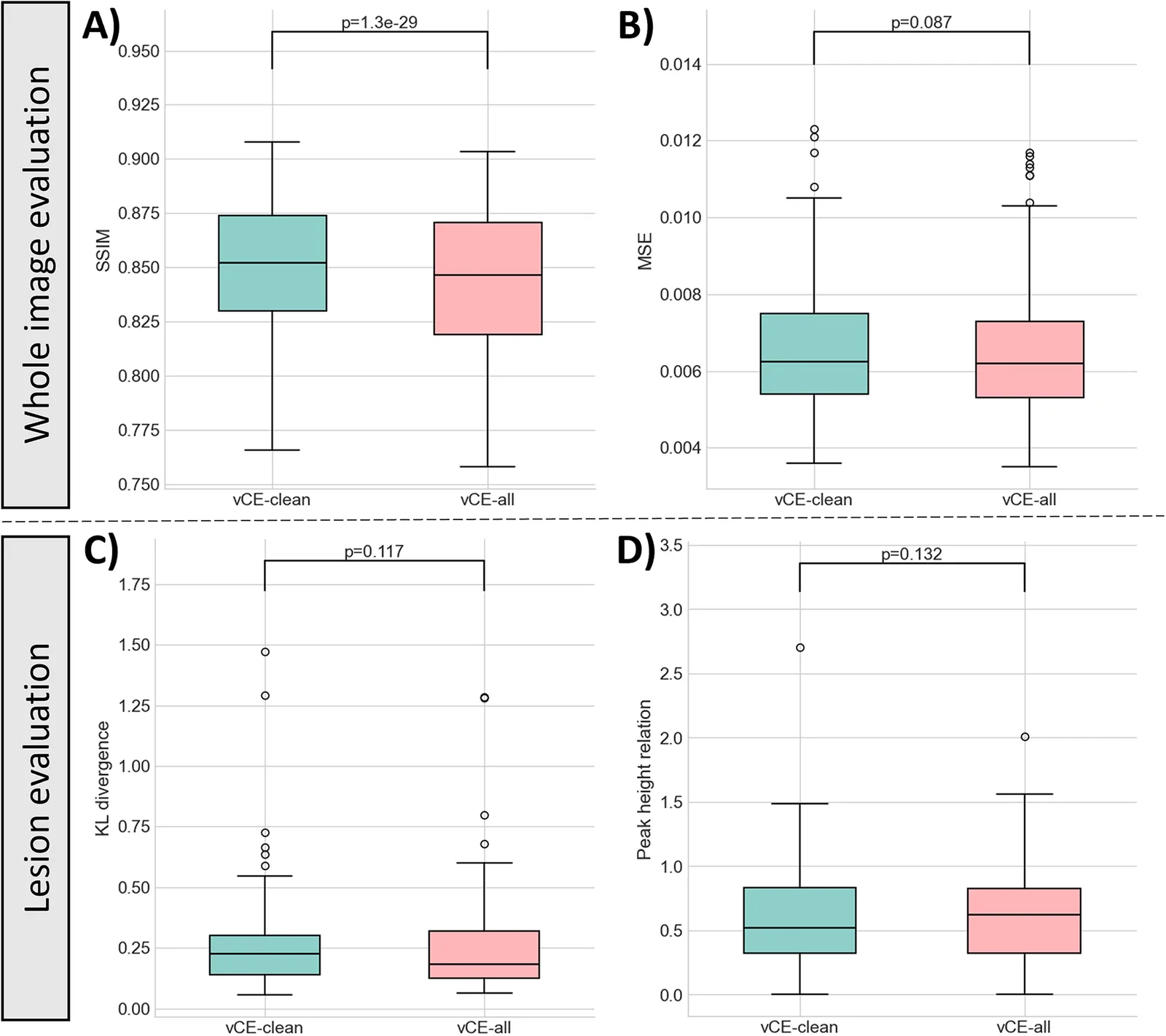
Boxplots of Quantitative Evaluation Metrics. The shown metrics compare the two virtual contrast-enhancement (vCE) approaches using artifact-reduced DWI data (vCE-clean) and the baseline approach using all available data (vCE-all). **A**) plots the SSIM of the whole image and shows a statistically significant difference between the two approaches. **B**) shows the MSE over the whole image with no statistical significance. **C**) and **D**) show metrics for the lesion segmentations. **C**) plots the KL divergence of the lesion area histograms with no statistically significant difference between approaches. **D**) shows the Peak height relation of the lesion area histograms with no statistically significant difference.

For the whole-image analysis on the hold-out test set, the mean SSIM was minimally higher for *vCE-clean* (0.849 ± 0.031) than for *vCE-all* (0.843 ± 0.034), corresponding to a mean paired difference of 0.0065 (95% CI: 0.0057–0.0073). Statistically, this difference was significant (*p* < 0.001). However, the standardized effect size was small (Cohen’s d_av_ = 0.20), indicating that the magnitude of the improvement was very limited. In contrast, no statistically significant difference was observed for MSE between the two networks (*vCE-clean*: 6.6 × 10⁻³ ± 1.6 × 10⁻³; *vCE-all*: 6.5 × 10⁻³ ± 1.7 × 10⁻³), with a mean paired difference of 6.1 × 10⁻⁵ (95% CI: −1.2 × 10⁻⁵ to 1.36 × 10⁻⁴; *p* = 0.087) and a negligible effect size (Cohen’s d_av_ = − 0.06). The corresponding distributions are shown in Fig. 4A) and 4B).

Lesion characteristics of the 61 cases with available segmentations are summarized in Table 2. For lesion-focused quantitative analysis, no statistically significant differences were observed between the networks. KL divergence of the lesion area histograms showed comparable values for *vCE-clean* (0.293 ± 0.258) and *vCE-all* (0.277 ± 0.249), corresponding to a mean paired difference of 0.017 (95% CI: −0.011 to 0.043), with no significant difference (*p* = 0.117) and a negligible effect size (Cohen’s d_av_ = 0.07), see Fig. 4C). Similarly, the lesion peak intensity relation of the lesion area histograms demonstrated no significant difference (*vCE-clean*: 0.615 ± 0.460; *vCE-all*: 0.630 ± 0.437), with a mean paired difference of − 0.015 (95% CI: −0.066 to 0.037; *p* = 0.132) and a negligible effect size (Cohen’s d_av_ = − 0.03), see Fig. 4D). Post-hoc normalized SSIM and MSE computed within lesion masks also did not differ significantly between the two networks (Supplementary Fig. 2), with negligible effect sizes for both lesion SSIM (Cohen’s d_av_ = − 0.06) and lesion MSE (Cohen’s d_av_ = − 0.06).

**Table 2:** Overview of lesion characteristics for the 61 cases included in the lesion-focused analysis. Reported parameters include patient age, lesion size, and BI-RADS classification. The BI-RADS score refers to the highest score given for an examination during routine clinical reading. Age is presented as the mean and standard deviation. Lesion volume is reported both as mean and standard deviation and as median with interquartile range (IQR) due to the skewed distribution

Metric	Number of lesions
61	Value
Age (years)	54.6 ± 11.4
Lesion volume (cm^3^)	6.71 ± 1.20 1.88 (IQR 0.92–6.34)
BI-RADS scores 4 5 6	7 15 39

### Qualitative analysis

The qualitative assessment of lesion enhancement revealed heterogeneous reader preferences. Two of three readers favored vCE-all in approximately half of the cases (46–51%), whereas the third reader rated vCE-clean and vCE-all equally often as better enhancing (19.67% each), see Table 3. Overall, lesion enhancement was rated as largely comparable between *vCE-clean* and *vCE-all.* Inter-reader agreement for lesion enhancement was low to moderate, with pairwise percent agreement ranging from 32% to 59% (R1. vs. R3 39.34%; R1 vs. R2. 59.02%; R3 vs. R2 32.79%). Pairwise Cohen’s κ values ranged from 0.106 to 0.380 (R1 vs. R3 κ = 0.181; R1 vs. R2 κ = 0.380; R3 vs. R2. κ = 0.106).

**Table 3:** Side-by-side comparison of lesion enhancement and image quality at artifact slices. Three independent readers (R1-3evaluated lesion enhancement in 61 lesion cases and image quality in 200 slices with confirmed severe DWI artifacts. Readers were asked to provide a preferred virtual contrast enhancement (vCE) image, blinded to the network used to generate the images

Reading	Reader	vCE-clean better	vCE-all better	Comparable	No lesion visibility
Lesion enhancement	R1	13 (21.31%)	31 (50.82%)	13(21.31%)	4 (6.56%)
	R2	18 (29.51%)	28 (45.90%)	11 (18.03%)	4 (6.56%)
	R3	12 (19.67%)	12 (19.67%)	32 (52.46%)	5 (8.2%)
Artifact slices	R1	92 (46%)	32 (16%)	76 (38%)	-
	R2	60 (30%)	99 (49.5%)	41 (20.5%)	-
	R3	49 (24.5%)	26 (13%)	125 (62.5%)	-

**Fig. 5:**
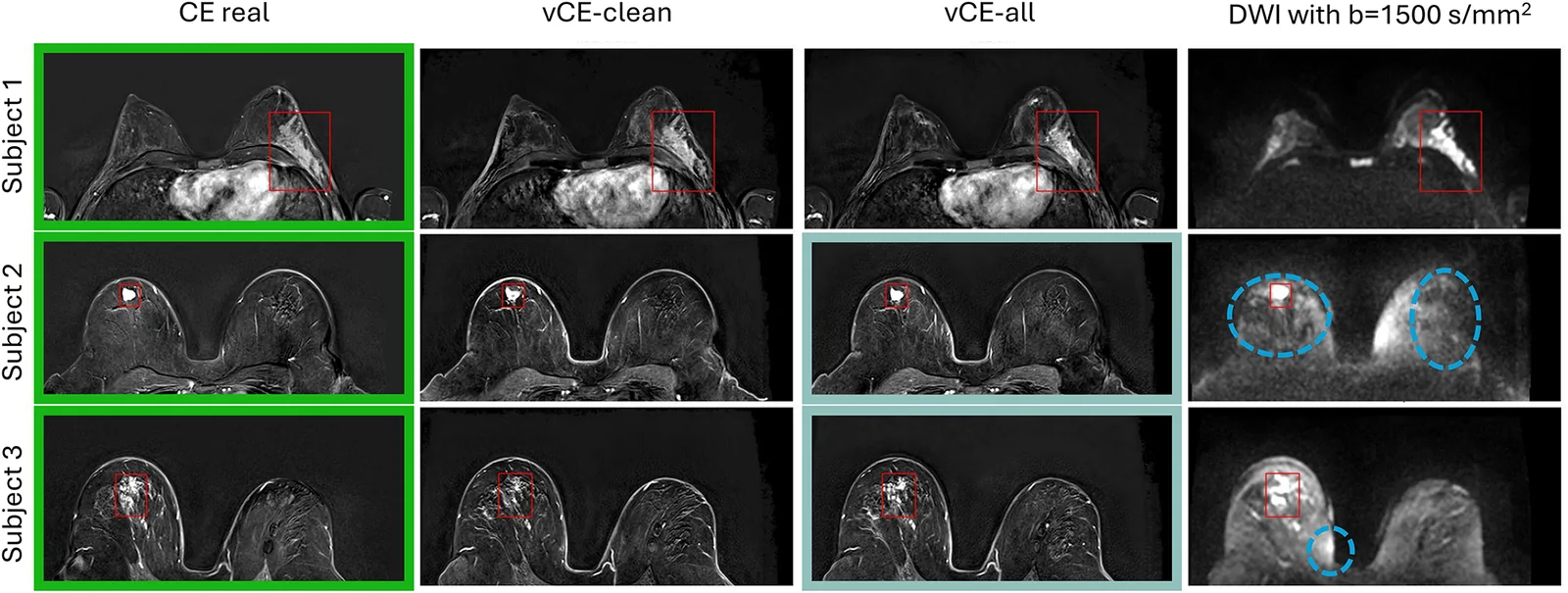
Representative slices of lesions from three subjects. For each subject, the original contrast-enhanced (CE) image, virtual contrast-enhanced images generated by the two networks (vCE-clean and vCE-all), and the corresponding diffusion-weighted image (DWI) at b = 1500 s/mm² are shown at the middle slice of the main lesion. Lesions are marked by red bounding boxes. Yellow dotted circles indicate diffusion intensity artifacts, including low-intensity (LI) artifacts (Subject 2) and high-intensity (HI) artifacts, likely related to surface coil effects (Subject 3).

Figure 5 presents representative middle slices of lesions from three subjects. For each subject, the original contrast-enhanced (CE) image, two versions of the vCE images (*vCE-clean* and *vCE-all*), and the corresponding diffusion-weighted image (DWI) at b = 1500 s/mm² are displayed. Lesions are indicated by red bounding boxes. In Subject 1, no visible artifacts are present in the DWI, and both vCE versions show similar lesion conspicuity according to all readers. For Subjects 2 and 3, all readers rated lesion visibility higher in *vCE-all* than in *vCE-clean*. Subject 2 shows LI artifacts in the DWI, while Subject 3 shows HI artifacts, likely related to surface coil effects, both highlighted by yellow dotted circles. Overall, the visual differences in lesion enhancement between the two networks are small.

In order to further investigate the influence of artifacts on DWI on the vCE images, specific slices with DWI artifacts were read with regard to the resulting vCE slices derived from the particular artifact-containing slice. Similar to the lesion enhancement task, no clear preference for one network was observed; see Table 3. While one reader favored *vCE-clean* in 46% of artifact-containing cases and another preferred *vCE-all* in 49.5%, the third reader showed a largely balanced distribution, with a slight tendency toward *vCE-clean* compared to *vCE-all* (24.5% vs. 13%) but overall comparable ratings for both networks. Inter-reader agreement in the artifact reading was again low to moderate, with pairwise percent agreement between 24% and 45% (R1 vs. R3 45.23%; R1 vs. R2 30.65%; R3 vs. R2 24.12%). Pairwise Cohen’s κ values ranged from − 0.035 to 0.134 (R1 vs. R3 κ = 0.134; R1 vs. R2 κ = 0.014; R3 vs. R2 κ = −0.035). This reflects pronounced variability in perceived image quality under artifact conditions. Notably, *vCE-clean*, despite not being trained on artifact-corrupted DWI inputs, maintained comparable image quality to *vCE-all* in many cases, indicating a certain degree of robustness to unseen artifact patterns.

### Correlation analysis

**Fig. 6:**
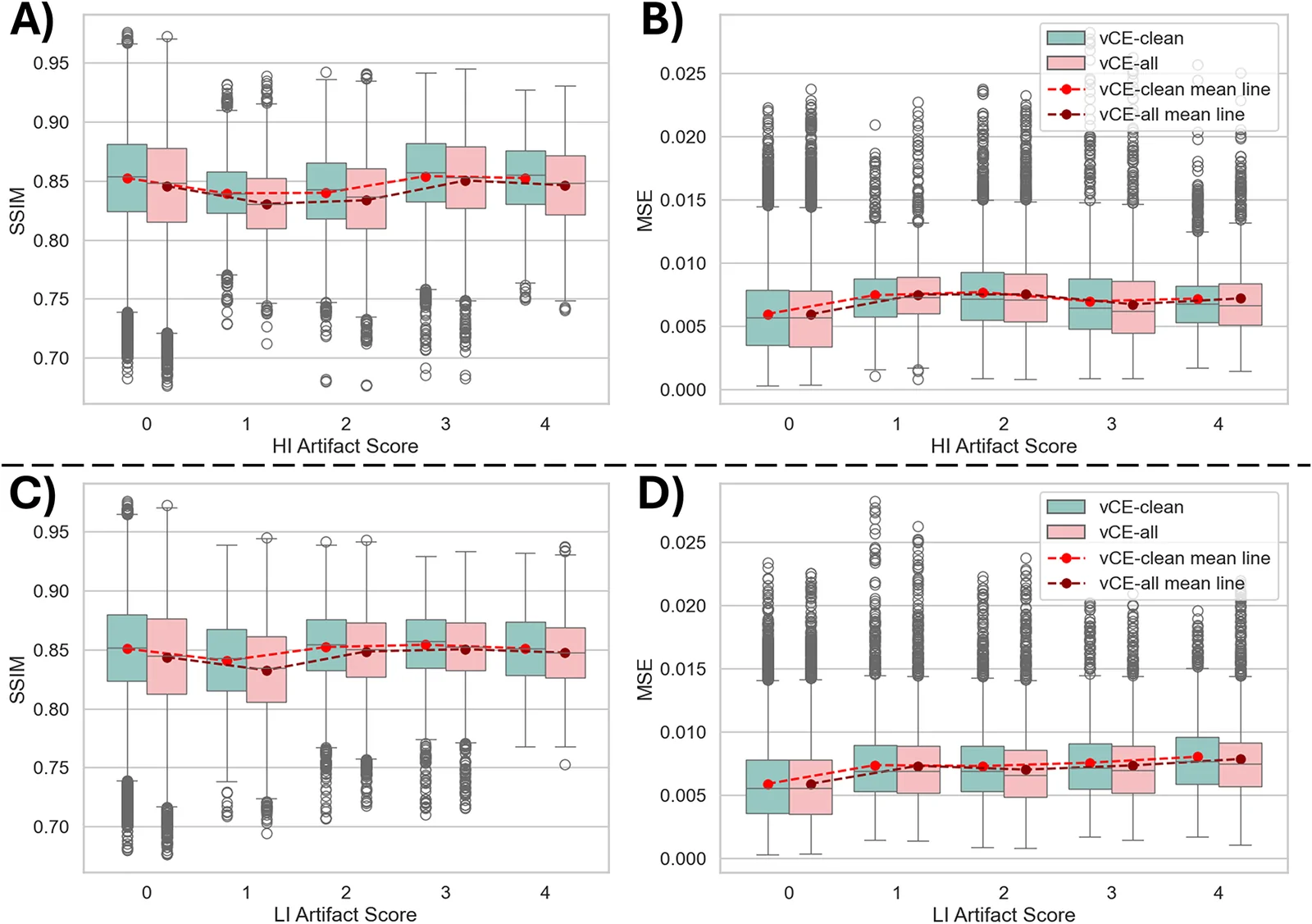
Whole-image performance metrics across artifact severity for both vCE network variants. **A**) SSIM versus high-intensity artifact score. **B**) MSE versus high-intensity artifact score. C) SSIM versus low-intensity artifact score. **D**) MSE versus low-intensity artifact score. Boxplots depict metric distributions per artifact category, and dotted lines indicate mean values per score for vCE-clean and vCE-all. Common legends are shown in panel (**B**) and (**D**).

For whole-image SSIM, no meaningful correlations with artifact score on high b-value DWI data were observed for either LI or HI artifacts and for neither vCE network variant. Spearman correlation coefficients were close to zero in all conditions (LI: vCE-clean ρ = 0.007, *p* = 0.291; vCE-all ρ = 0.022, *p* < 0.001; HI: vCE-clean ρ = 0.037, *p* < 0.001; vCE-all ρ = 0.024, *p* < 0.001), indicating that whole-image SSIM was largely insensitive to increasing artifact severity in DWI inputs. This trend is also shown in Fig. 6A) and 6 C). Whole-image MSE showed small positive correlations with artifact score for both intensity type artifacts and for both network variants (LI: vCE-clean ρ = 0.260, *p* < 0.001; vCE-all ρ = 0.218, *p* < 0.001; HI: vCE-clean ρ = 0.197, *p* < 0.001; vCE-all ρ = 0.179, *p* < 0.001), see Fig. 6B) and D). Although most correlations reached statistical significance (*p* < 0.001), likely due to the large number of slice-wise observations, effect sizes remained small. Given the strong imbalance of artifact scores toward score 0 and the presence of multiple slices per patient, p-values were interpreted with caution and were not considered indicative of clinically relevant effects. Detailed p-values for the whole correlation analysis are presented in Supplemental Table 2.

When restricting the analysis to slices with artifact scores greater than zero, correlations remained weak. For SSIM, very small positive correlations were observed for both artifact intensities and both networks (LI: *vCE-clean* ρ = 0.128, *vCE-all* ρ = 0.169; HI: *vCE-clean* ρ = 0.192, *vCE-all* ρ = 0.217). For MSE, correlations were negligible for LI artifacts (*vCE-clean* ρ = 0.070, *vCE-all* ρ = 0.036) and weakly negative for HI artifacts (*vCE-clean* ρ = −0.094, *vCE-all* ρ = −0.125), indicating no consistent degradation of whole-image MSE with increasing artifact severity once artifact-free slices were excluded. All correlations were statistically significant (*p* < 0.001).

Overall, whole-image metrics showed limited sensitivity to artifact severity, particularly when considering only artifact-affected slices.

For lesion-based analyses, no statistically significant correlations were observed between artifact percentage within lesion slices and lesion peak-height relation for either artifact type, network variant, or analysis condition (including or excluding lesions without strong artifacts, all p-values > 0.05). Although small Spearman correlation coefficients were observed in some cases, none reached statistical significance (see Table 4 and Supplemental Table 2). In contrast, weak negative correlations were observed between artifact percentage and KL divergence of lesion area histograms for HI artifacts when all lesions were included (vCE-clean: Spearman ρ = −0.288, *p* = 0.025; vCE-all: ρ = −0.253, *p* = 0.049). However, these associations were not reproduced in the restricted analysis excluding lesions without strong-artifact-affected slices (all p-values > 0.05). Consistently, ANCOVA models with interaction terms revealed no significant interaction (*p* > 0.05) between artifact percentage and network variant, indicating no differential sensitivity of the two networks with respect to these lesion-based metrics.

**Fig. 7:**
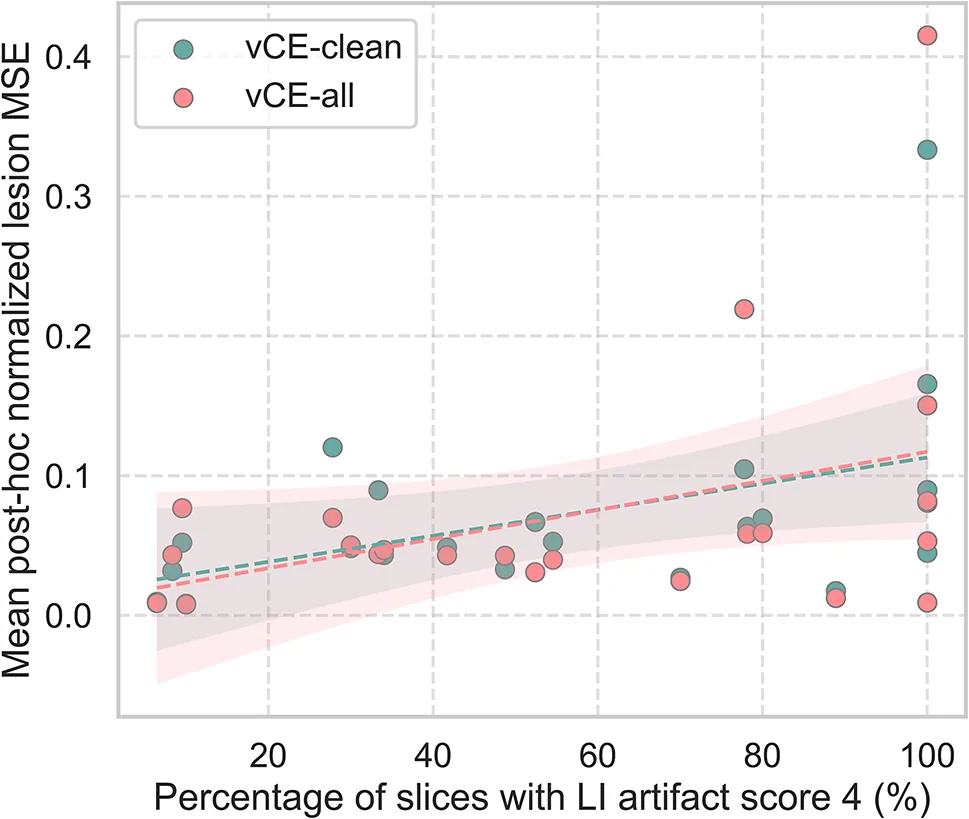
Correlation of mean post-hoc normalized MSE inside the lesion and severe hypointense artifact (score = 4) percentage of lesion slices. Mean post-hoc normalized lesion MSE is shown as a function of the percentage of lesion slices affected by hypointense artifact score 4, excluding lesions with 0% artifact involvement. Each point corresponds to one lesion, with different colors for the two different networks, vCE-clean and vCE-all, and with separate trend lines and shaded areas representing the corresponding 95% confidence intervals. Statistical associations were evaluated using Spearman's rank correlation coefficient.

In contrast, post-hoc normalized lesion MSE showed a moderate positive association with artifact percentage for LI artifacts in the *vCE-clean* network when restricting the analysis to lesions with only strong-artifact-affected lesions (*n* = 12 cases) (Spearman ρ = 0.478, *p* = 0.025), noting that this analysis was based on a very small subset, as shown in Fig. 7. In addition, when all lesions were included, a weak negative correlation was observed for HI artifacts in ​both networks with the post-hoc normalized lesion MSE (*vCE-*all: ρ = −0.379, *p* = 0.003; *vCE-*clean: ρ = −0.398, *p* = 0.001). However, these associations were not reproduced in the restricted analysis excluding lesions without strong-artifact-affected slices. Because most lesions had 0% strong-artifact-affected slices, resulting in a highly skewed distribution of artifact percentages, and because this finding was isolated and of small magnitude, it should be interpreted with caution. For the remaining conditions no statistically significant correlations were observed.

ANCOVA analysis for LI artifacts confirmed a significant main effect of artifact percentage on post-hoc normalized lesion MSE (*p* = 0.037), while neither network version (*p* = 0.84) nor the interaction between artifact percentage and network version (*p* = 0.88) was significant. These findings indicate that, for LI artifacts, increasing artifact strength is associated with higher post-hoc normalized lesion MSE, whereas the choice of vCE network did not significantly modify this relationship.

Detailed Spearman correlation coefficients for all metrics, artifact type, and network combinations can be found in Table 4. Overall, whole-image metrics were largely insensitive to artifact severity. For lesion-based metrics, significant associations were primarily observed in analyses including all lesions for HI artifacts, whereas these associations disappeared after restricting the analysis to lesions containing strong-artifact-affected slices. In contrast, post-hoc normalized lesion MSE showed a positive association with increasing LI artifact burden in the restricted analysis, suggesting that LI artifacts may have a stronger negative impact on lesion reconstruction quality in directly affected lesions.

**Table 4:** Spearman correlation coefficients (ρ) between artifact measures and vCE performance metrics for whole-image and lesion-based analyses. Whole-image metrics (SSIM, MSE) were correlated slice-wise with the corresponding artifact score of each slice. Lesion-based metrics (KL divergence, lesion peak-height relation, post-hoc normalized lesion MSE) were correlated lesion-wise with the percentage of artifact-affected slices within each lesion. Correlations are reported for all samples and for restricted analyses excluding artifact-free cases (artifact score > 0 for whole-image metrics; strong-artifact-affected slice percentage > 0% for lesion metrics). Statistical significance is indicated by * (*p* < 0.05); n.s. denotes non-significant results. Correlation analyses should be interpreted as exploratory because several subgroup analyses were based on relatively small sample sizes

Whole image metrics				
Metric	Artifact type	Network	All scores ρ	Scores > 0, ρ
SSIM (whole)	LI	*vCE-clean*	0.007	0.128
SSIM (whole)	LI	*vCE-all*	0.022	0.169
SSIM (whole)	HI	*vCE-clean*	0.037	0.192
SSIM (whole)	HI	*vCE-all*	0.024	0.217
MSE (whole)	LI	*vCE-clean*	0.260	0.070
MSE (whole)	LI	*vCE-all*	0.218	0.036
MSE (whole)	HI	*vCE-clean*	0.197	-0.094
MSE (whole)	HI	*vCE-all*	0.179	-0.125
**Lesion metrics**				
**Metric**	**Artifact type**	**Network**	**All lesions**,** ρ**	**Artifact score > 0%**,** ρ**
KL divergence	LI	*vCE-clean*	-0.110, n.s.	0.198, n.s.
KL divergence	LI	*vCE-all*	-0.188, n.s.	0.190, n.s.
KL divergence	HI	*vCE-clean*	-0.288, *	0.056, n.s.
KL divergence	HI	*vCE-all*	-0.253, *	-0.263, n.s.
Peak height relation	LI	*vCE-clean*	0.040, n.s.	-0.264, n.s.
Peak height relation	LI	*vCE-all*	-0.136, n.s.	-0.222, n.s.
Peak height relation	HI	*vCE-clean*	0.159, n.s.	0.014, n.s.
Peak height relation	HI	*vCE-all*	0.186, n.s.	0.011, n.s.
MSE (lesion)	LI	*vCE-clean*	-0.117, n.s.	0.478, *
MSE (lesion)	LI	*vCE-all*	-0.189, n.s.	0.324, n.s.
MSE (lesion)	HI	*vCE-clean*	-0.398, *	0.084, n.s.
MSE (lesion)	HI	*vCE-all*	-0.379, *	-0.028, n.s.

### Exploratory scanner-stratified analysis

Scanner-specific analysis revealed significant differences in the distribution of DWI artifacts between the two MRI systems (Supplementary Fig. 3). Compared with the MAGNETOM Skyra Fit, examinations acquired on the MAGNETOM Vida exhibited a lower prevalence of mild HI artifacts (class 1: 11.8% vs. 4.9%; class 2: 14.6% vs. 9.6%), but a higher prevalence of moderate-to-severe HI artifacts (class 3: 10.5% vs. 19.3%). Similarly, for LI artifacts, the MAGNETOM Skyra Fit showed a higher proportion of mild artifacts (class 0: 64.9% vs.58.2%; class 1: 23.0% vs. 10.4%), whereas the MAGNETOM Vida exhibited a higher prevalence of moderate-to-severe artifacts (classes 2–4: 31,4% vs. 12.1%). Examination-level comparisons confirmed significant differences in artifact distributions for all LI artifact classes and for HI artifact classes 1–3 after Holm correction for multiple comparisons.

For whole-image SSIM, vCE-clean achieved significantly higher values than vCE-all on both MRI systems (MAGNETOM Vida: 0.868 ± 0.021 vs. 0.865 ± 0.021, *p* < 0.001; MAGNETOM Skyra Fit: 0.825 ± 0.025 vs. 0.813 ± 0.024, *p* < 0.001). The improvement was more pronounced for the MAGNETOM Skyra Fit (ΔSSIM = 0.0115) than for the MAGNETOM Vida (ΔSSIM = 0.0028). Independent of the model, examinations acquired on the MAGNETOM Vida demonstrated significantly higher whole-image SSIM than those acquired on the MAGNETOM Skyra Fit (both *p* < 0.001).

For whole-image MSE, vCE-all achieved slightly lower values than vCE-clean on the MAGNETOM Vida (0.0062 ± 0.0017 vs. 0.0065 ± 0.0017, *p* < 0.001), whereas vCE-clean yielded slightly lower MSE than vCE-all on the MAGNETOM Skyra Fit (0.0067 ± 0.0015 vs. 0.0070 ± 0.0016, *p* < 0.001). Between scanners, no significant difference in MSE was observed for vCE-clean (*p* = 0.113), whereas vCE-all demonstrated significantly lower MSE on the MAGNETOM Vida than on the MAGNETOM Skyra Fit (*p* < 0.001).

In contrast, lesion-based performance was largely consistent across scanners. Neither lesion SSIM nor lesion MSE differed significantly between vCE-clean and vCE-all within either scanner subgroup, and no significant scanner-specific differences were observed for lesion SSIM. More detailed information for the scanner-stratified analysis for whole-image and lesion-based metrics can be found in Supplementary Tables 3 and Supplementary Fig. 4.

## Discussion

This study investigated how the presence of strong high- and low-intensity (HI and LI) artifacts in high b-value DWI images affects the performance of neural networks generating virtual contrast-enhanced (vCE) images from unenhanced breast MRI datasets. Given recent evidence that high b-value DWI may represent one of the most influential input sequences for enhancement synthesis [19], we specifically focused on whether degradation of this dominant input source affects global image quality and lesion depiction differently.

Our results demonstrated that while excluding DWI intensity artifact-affected slices during training led to slightly higher yet statistically significant whole-image structural similarity, no relevant differences were observed in pixel-wise error or qualitative assessment results of lesion enhancement and image quality, suggesting that the statistically significant improvement was of limited practical relevance.

As lesion-based quantitative metrics showed similarly no meaningful differences between the two networks, it might be interpreted that both models learned lesion enhancement characteristics equally well and that training without strongly artifact-affected DWI slices does not substantially improve or worsen vCE performance in lesion enhancement.

Thus, although DWI has been suggested to be highly informative for enhancement synthesis, the performance of the network was only slightly affected by the exclusion of artifact-affected slices under the investigated conditions. This was further supported by the qualitative reader assessment, in which no consistent preference for either network output was observed across readers. The low pairwise inter-reader agreement (Cohen’s κ ranging from − 0.035 to 0.134 for artifact-related preference) further indicates that the preferred output varied substantially between readers. Together, these findings suggest that the outputs of the different network training approaches were largely interchangeable in terms of visually preferable imaging characteristics, with neither approach showing a consistent qualitative advantage.

Correlation analysis showed that whole-image similarity metrics were largely insensitive to artifact severity in the input DWI.

For lesion-based analyses, significant negative correlations between artifact percentage and both post-hoc normalized lesion MSE and KL divergence were initially observed for HI artifacts only when all lesions were included. However, these associations disappeared after restricting the analysis to lesions containing at least one strong-artifact-affected slice. Because the majority of lesions contained no strong-artifact-affected slices, resulting in a highly skewed distribution of artifact percentages, these findings should be interpreted with caution and are unlikely to reflect a clinically meaningful effect of increasing HI artifact burden. In contrast, post-hoc normalized lesion-based MSE increased with increasing severity of LI artifacts, while no such association was observed for HI artifacts. This suggests that LI artifacts may have a stronger negative impact on lesion enhancement in virtual contrast images. However, given the generally weak correlations and exploratory nature of the analyses, no definitive conclusions regarding the clinical impact can be drawn.

From a diffusion MRI perspective, LI artifacts represent localized signal loss, resulting in the absence of information within affected regions. Because diffusion restriction has been reported to provide relevant imaging characteristics of breast lesions, loss of this signal may reduce the amount of lesion-specific information available to the network for enhancement synthesis. In contrast, HI artifacts introduce additional noise signal rather than removing existing tissue information completely. Although such signal elevations may potentially induce spurious enhancement patterns, underlying anatomical structures often remain at least partially visible, allowing the network to extract relevant contextual information. From a deep learning perspective, the generator relies on local image features and contextual relationships learned during training. Complete signal voids caused by LI artifacts may therefore disrupt feature extraction more severely than signal increases, as relevant lesion characteristics are no longer represented in the input image. These findings support the concept that the effect of DWI quality is more pronounced locally at the lesion level than globally across the entire synthesized image. However, correlation analyses were performed without adjustment for multiple testing, and several subgroup analyses were based on small sample sizes.

Further, it should be considered that a relatively low interobserver agreement was noticed for the qualitative features, which may, in part, be explained by the heterogeneous breast MRI experience of the three readers (0.5, 5, and > 10 years), as differences in experience can influence the assessment of subtle image quality characteristics and lesion enhancement.

Using artificial intelligence (AI) algorithms for deriving vCE images is an emerging research area that is of special interest for the field of breast MRI. Previous studies have indicated the principal feasibility; however, they frequently only included T1w and T2w sequences [20]. More recent work has shown that including diffusion-weighted imaging (DWI) during training significantly contributes to vCE performance, especially with respect to lesion depiction. In line with this, Liebert et al. reported that DWI has a limited impact on overall image quality compared to structural sequences such as T2-weighted imaging, but plays a more important role in lesion enhancement [19]. Our findings complement prior sequence-ablation studies by showing that not only the presence of DWI, but also its image quality, may be relevant for reliable lesion representation in vCE imaging.

The present study employed the previously validated pix2pix GAN architecture by George et al., which demonstrated improved visual realism compared with a conventional U-Net in breast vCE synthesis [17]. Accordingly, the focus of this work was not to compare network architectures, but to investigate the influence of DWI artifacts on the performance of this established vCE framework.

DWI is more prone to developing image artifacts than other MRI sequences, and thus is at risk of having limited diagnostic image quality. DWI artifacts can originate from a range of causes, including insufficient fat saturation and some signal voids, which are attributed to, e.g., pulsation artifacts. This results in different types of artifacts, which can be either signal-hyperintense areas, obscuring the original structures of the breast, or result in hypointense artifacts, voiding any remaining tissue signal [25–27].

Our study did indicate that hypointense artifacts might be more challenging if present during training than hyperintense artifacts.

The evaluation of vCE images remains challenging, as no single metric adequately reflects both global image fidelity and clinically relevant lesion characteristics. In this study, whole-image similarity metrics were used to assess global structural and intensity agreement, while lesion-based metrics and qualitative reader assessments were employed to evaluate lesion-specific enhancement and perceived diagnostic quality. This combined strategy was chosen to capture both technical image similarity and clinically meaningful differences. Importantly, no universally accepted threshold exists for a clinically perceptible or diagnostically meaningful difference in SSIM for breast MRI or virtual contrast-enhanced image synthesis. Direct comparison of SSIM values across studies should be interpreted with caution, as SSIM depends on factors including the underlying dataset, image preprocessing, prediction target, and evaluation protocol. Therefore, the small but statistically significant increase in whole-image SSIM should not be interpreted in isolation but rather in conjunction with the lesion-based quantitative analyses, qualitative reader assessment, and the small standardized effect size, all of which indicated comparable performance between the two networks.

Osuala et al. reported SSIM values for single time‑point DCE subtraction image prediction using a GAN ranging from 0.701 to 0.765 across different time points, without evaluation of lesion-specific performance [20]. Our network achieved an SSIM of approximately 0.84, indicating competitive global image similarity within the present experimental setting. George et al., whose GAN network served as the basis for our vCE approach, reported an SSIM of ~ 0.80 for the GAN and 0.87 for a U‑Net, while noting that the GAN produced qualitatively more realistic images [17]. A similar pattern is observed in our study: *vCE-clean* shows a slight but statistically significant improvement in SSIM compared to *vCE-all*, yet visual assessment indicated no meaningful differences in image quality, at least for slices affected by DWI artifacts. These results suggest that, while our network achieves competitive quantitative performance, subtle differences in qualitative image appearance remain, consistent with prior observations in GAN- and U‑Net-based vCE networks.

Our study has several limitations. The study cohort included a retrospective dataset only, and no prospective evaluation has been performed. Furthermore, although data were acquired on two clinical 3 T MRI systems, both scanners originated from the same vendor and a single institution, limiting the external generalizability of our findings. Exploratory scanner-stratified analyses demonstrated differences in whole-image performance between the two scanners, whereas lesion-based performance remained largely comparable. However, substantially more MAGNETOM Vida examinations were available for model training than MAGNETOM Skyra Fit examinations, which may have biased the network toward scanner-specific image characteristics. Because scanner type was not experimentally balanced, the independent contribution of scanner representation cannot be determined. Therefore, external validation across institutions, vendors, field strengths, and acquisition protocols remains necessary. Moreover, Scanner-specific differences in DWI artifact prevalence were also observed, with severe LI and HI artifacts occurring more frequently on the MAGNETOM Vida. These findings suggest that scanner-dependent differences in vCE performance cannot be explained solely by artifact prevalence and may additionally reflect differences in acquisition characteristics, image reconstruction, or image contrast. Further, artifact severity was determined using an automatic artifact detection network, which is itself subject to classification errors. Misclassification of artifact strength may have influenced the observed correlations between artifact severity and vCE performance and reduced the sensitivity of the analysis. Furthermore, only the exclusion of slices with the highest artifact score was investigated, and given the small effect size at significant signal voids, further experiments in minimal/moderate artifacts were not performed.

Additionally, the study only investigated the apparent type of image artifact in terms of HI and LI without further stratifying the origin of the artifact. This might be subject to future projects in order to better understand the influence of subclasses and the technical origin on the image quality in subsequent image processing. Additionally, we did not include only datasets where the image artifacts factually obscured lesions. Thus, we cannot draw final conclusions about whether the artifacts would have prevented a lesion present from being visualized on the vCE images.

From a translational perspective, the present findings should be interpreted with caution. Since the study was not restricted to cases in which DWI artifacts directly obscured lesions, no definitive conclusions can be drawn regarding the clinical impact of these artifacts on vCE image interpretation. It is conceivable that LI artifacts affecting lesion regions may have a greater influence on synthesized lesion appearance than reflected in the present cohort. Therefore, future studies should specifically investigate cases in which lesions are directly affected by image artifacts. Furthermore, automated detection of poor DWI image quality or severe artifacts during or immediately after image acquisition and before vCE synthesis could represent a useful quality-control step within future AI-augmented (semi-)automated imaging workflows. Such an approach could enable artifact-affected acquisitions to be flagged at an early stage and potentially prompt reacquisition when appropriate. Although the overall influence of artifact exclusion during training was limited in this study, identifying severely artifact-affected examinations may improve network robustness and help flag cases requiring careful interpretation or additional imaging. In addition, future work should investigate whether HI artifacts may introduce misleading enhancement patterns or other image inconsistencies during vCE synthesis, as such effects could influence radiologist interpretation even if quantitative image quality metrics remain largely unchanged.

Finally, although qualitative image quality and lesion enhancement were assessed by human readers, we did not investigate whether the observed differences in vCE image quality influence BI-RADS assessment, diagnostic confidence, or subsequent clinical decision-making. Future studies should determine whether artifact-related changes in vCE synthesis translate into clinically meaningful differences in lesion detection, characterization, and patient management.

Further, artifact analysis was restricted to high b-value DWI images, although the vCE network uses multiple input sequences. The DWI protocol used in this study, comprising three b-values, was determined by the available dataset and is consistent with previous work demonstrating that incorporating multi-b-value DWI together with morphological T1-weighted and T2-weighted images substantially improves vCE image quality compared with models without multi-b-value DWI [19]. Nevertheless, different DWI acquisition protocols, including alternative numbers or combinations of b-values, may alter tissue contrast, signal-to-noise ratio, lesion conspicuity, and artifact characteristics. Consequently, the contribution of DWI to vCE generation, as well as the impact of image degradation, may differ across protocols. Artifacts in other sequences, particularly other b-values in DWI, T2-weighted images, or pre-contrast T1 images, may also influence vCE quality and lesion depiction [42]. Consequently, the analyses performed here can only demonstrate correlations and do not allow conclusions about causality between specific artifact types and vCE performance.

Additionally, we only investigated a single neural network for generating the vCE; however, other networks, such as U-Net or CycleGAN approaches, could be influenced to varying degrees given their different architectural designs [43, 44].

## Conclusion

In conclusion, image artifacts on DWI images influenced neural networks for generating virtual contrast-enhanced images during inference, with low-signal intensity artifacts showing a higher impact than high-signal artifact areas. However, excluding artifact-affected slices during training did not lead to relevant improvements in visual image quality or lesion depiction, and instead demonstrated largely comparable performance to training on the whole dataset. These findings suggest that the investigated vCE model appeared relatively robust to the exclusion of strongly artifact-affected training slices, but severe degradation of the dominant DWI input sequence may still compromise lesion conspicuity during deployment. Given the high prevalence of artifacts on DWI and its significance for virtual contrast-enhancement, further research is needed to understand the implications for virtual contrast-enhanced imaging in clinical routine.

## Supplementary Information

Below is the link to the electronic supplementary material.

Supplementary Material 1

Supplementary Material 2

Supplementary Material 3

Supplementary Material 4

Supplementary Material 5

Supplementary Material 6

Supplementary Material 7

## Data Availability

The datasets generated and analyzed during the current study, as well as the associated model weights, are not publicly available due to restrictions imposed by the General Data Protection Regulation (GDPR/DSGVO) and national data protection laws but are available from the corresponding author on reasonable request.

## Abbreviations

ADC: Apparent diffusion coefficient
AI: Artificial intelligence
BCa: Bias-corrected and accelerated
BI-RADS: Breast imaging-reporting and data system
BMI: Body mass index
CE: Contrast-enhanced
CI: Confidence interval
DCE: Dynamic contrast-enhanced
DWI: Diffusion-weighted image
EUSOBI: European society of breast imaging
FoV: Field-of-view
GBCA: Gadolinium-based contrast agents
HI: High-intensity
IQR: Interquartile range
KL: Kullback-leibler
LI: Low-intensity
MRI: Magnetic resonance imaging
MSE: Mean squared error
n.s.: no statistical significance
SSIM: Structural similarity index measure
TR: Repetition time
TE: Echo time
T1w: T1-weighted
T2w: T2-weighted
vCE: Virtual ​contrast enhancement
vDCE: Virtual dynamic contrast enhancement

## Abbreviations

ADC: Apparent diffusion coefficient
AI: Artificial intelligence
BCa: Bias-corrected and accelerated
BI-RADS: Breast imaging-reporting and data system
BMI: Body mass index
CE: Contrast-enhanced
CI: Confidence interval
DCE: Dynamic contrast-enhanced
DWI: Diffusion-weighted image
EUSOBI: European society of breast imaging
FoV: Field-of-view
GBCA: Gadolinium-based contrast agents
HI: High-intensity
IQR: Interquartile range
KL: Kullback-leibler
LI: Low-intensity
MRI: Magnetic resonance imaging
MSE: Mean squared error
n.s.: no statistical significance
SSIM: Structural similarity index measure
TR: Repetition time
TE: Echo time
T1w: T1-weighted
T2w: T2-weighted
vCE: Virtual contrast enhancement
vDCE: Virtual dynamic contrast enhancement
