# Supplementary material for: Influence of dominant sequence artifacts on generative AI for virtual contrast-enhancement in breast MRI
Source: BMC Med Imaging. 2026 Nov 3;26:578. doi: 10.1186/s12880-026-02996-8 (PMC13729665; doi:10.1186/s12880-026-02996-8)
Supplement: Supplementary file 1 [file 12880_2026_2996_MOESM1_ESM.pdf]

S1 Table. MRI protocol. FoV field of view, TR repetition time, TE echo time, IR inversion recovery time, DWI diffusion-weighted imaging, GRE gradient echo, SE spin echo, EPI echo-planar imaging.

Sequence	Sequence Type	Matrix size	FoV (mm x mm)	Slice thickness (mm)	TR (ms)	TE (ms)	IR (ms)	FA (°)	No. of averages
T1w[a]	3D-GRE	448 x 448 x 112-144	360-420 × 360-420	1.5–1.8	5.51-5.97	2.46	-	8-10	1
T2w	2D-SE	447-512 × 448-512 × 34-76	340-400 × 340-400	4	3570-5670	60-70	230	60-108	1-2
DWI[b]	2D-IR-DWI-EPI	160-208 × 256 × 34-46	340-420 × 218-284	4	5810-13440	66-80	220-250	90	3/8/20 or 3/8/15[c]

(a) Acquired before and during the five time points after intravenous GBCA injection (gadobutrol; Bayer, Leverkusen, Germany; 0.1 mmol/kg/body weight, injection speed = 2 mL/s). Acquisitions were performed in 60/70 second intervals, with the acquisition of the DCE images starting 20/25 seconds after the administration of the GBCA.

(b) DWI scan was acquired using three b-values: 50, 750, and 1500 s/mm^2.

(c) No. of averages for DWI indicates the number of averages for each b-value. The first set of values was used in acquisitions performed on both Skyra-Fit and MAGNETOM VIDA and the second set was used only for acquisitions performed on MAGNETOM VIDA.
