# Supplementary material for: Influence of dominant sequence artifacts on generative AI for virtual contrast-enhancement in breast MRI
Source: BMC Med Imaging. 2026 Nov 3;26:578. doi: 10.1186/s12880-026-02996-8 (PMC13729665; doi:10.1186/s12880-026-02996-8)
Supplement: Supplementary file 2 [file 12880_2026_2996_MOESM2_ESM.pdf]

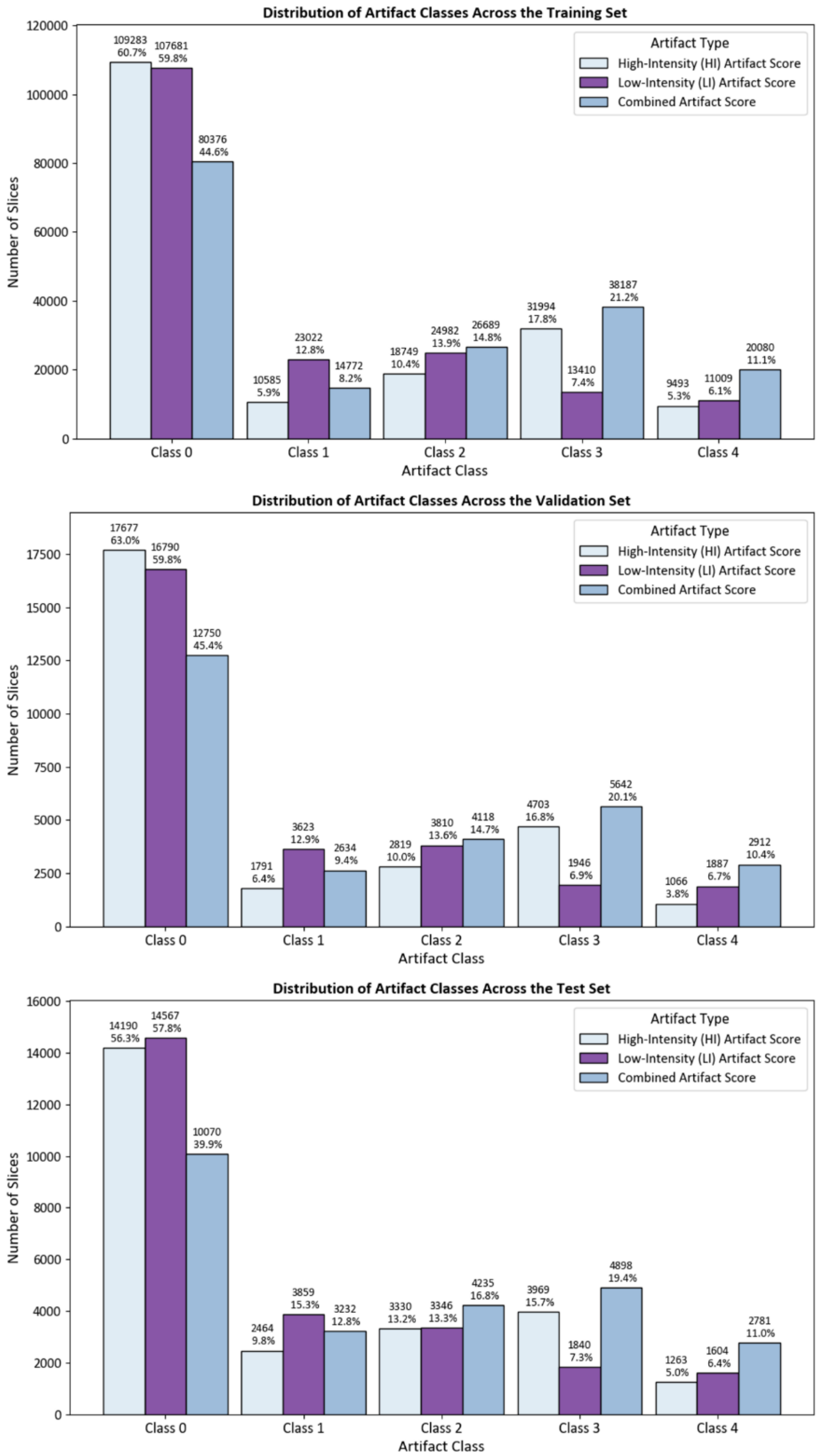

S1 Fig. Distribution of DWI intensity artifact severity across slices per Dataset. For the training, validation and test set, artifact class distributions are shown for the high-intensity (HI) model, low-intensity (LI) model, and the combined artifact score obtained by selecting the higher of the two scores. The majority of slices were artifact-free (Class 0), while severe artifacts (Class 4) were relatively rare. Moderate artifact levels (Classes 2–3) constituted a relevant fraction of the data, indicating clinically meaningful artifact burden.
