# Supplementary material for: Influence of dominant sequence artifacts on generative AI for virtual contrast-enhancement in breast MRI
Source: BMC Med Imaging. 2026 Nov 3;26:578. doi: 10.1186/s12880-026-02996-8 (PMC13729665; doi:10.1186/s12880-026-02996-8)
Supplement: Supplementary file 3 [file 12880_2026_2996_MOESM3_ESM.pdf]

S2 Table. P-values of Spearman correlation coefficients (ρ) analysis between artifact measures and vCE performance metrics for whole-image and lesion-based analyses. Whole-image metrics (SSIM, MSE) were correlated slice-wise with the corresponding artifact score of each slice. Lesion-based metrics (KL divergence, lesion peak-height relation, lesion MSE) were correlated lesion-wise with the percentage of artifact-affected slices within each lesion. Correlations are reported for all samples and for restricted analyses excluding artifact-free cases (artifact score > 0 for whole-image metrics; strong-artifact-affected slice percentage > 0% for lesion metrics).

p-values of whole image metrics

Metric	Artifact type	Network	All scores	Scores > 0
SSIM (whole)	LI	*vCE-clean*	0.291	<0.001
SSIM (whole)	LI	*vCE-all*	<0.001	<0.001
SSIM (whole)	HI	*vCE-clean*	<0.001	<0.001
SSIM (whole)	HI	*vCE-all*	<0.001	<0.001
MSE (whole)	LI	*vCE-clean*	<0.001	<0.001
MSE (whole)	LI	*vCE-all*	<0.001	<0.001
MSE (whole)	HI	*vCE-clean*	<0.001	<0.001
MSE (whole)	HI	*vCE-all*	<0.001	<0.001

p-values of lesion metrics

Metric	Artifact type	Network	All lesions	Artifact score > 0%
KL divergence	LI	*vCE-clean*	0.398	0.377
KL divergence	LI	*vCE-all*	0.147	0.396
KL divergence	HI	*vCE-clean*	**0.025**	0.863
KL divergence	HI	*vCE-all*	**0.049**	0.409
Peak height relation	LI	*vCE-clean*	0.757	0.235
Peak height relation	LI	*vCE-all*	0.296	0.321
Peak height relation	HI	*vCE-clean*	0.222	0.966

Peak height relation	HI	*vCE-all*	0.151	0.974
MSE (lesion)	LI	*vCE-clean*	0.371	**0.025**
MSE (lesion)	LI	*vCE-all*	0.144	0.141
MSE (lesion)	HI	*vCE-clean*	**0.001**	0.795
MSE (lesion)	HI	*vCE-all*	**0.003**	0.931
