# Supplementary material for: Influence of dominant sequence artifacts on generative AI for virtual contrast-enhancement in breast MRI
Source: BMC Med Imaging. 2026 Nov 3;26:578. doi: 10.1186/s12880-026-02996-8 (PMC13729665; doi:10.1186/s12880-026-02996-8)
Supplement: Supplementary file 4 [file 12880_2026_2996_MOESM4_ESM.pdf]

S3 Table. Scanner-stratified quantitative performance of the virtual contrast-enhanced (vCE) models. Whole-image and lesion-based structural similarity index (SSIM) and mean squared error (MSE) are reported separately for examinations acquired on the MAGNETOM Vida and MAGNETOM Skyra Fit MRI systems. Values are presented as mean ± standard deviation. Paired Wilcoxon signed-rank tests were used to compare vCE-clean and vCE-all within each scanner subgroup. Scanner-specific differences for each model were assessed using Mann–Whitney U tests.

Scanner-stratified performance of the vCE models

**Metric**	**Scanner**	***vCE-clean***	***vCE-all***	**p-value**
Whole-image SSIM	Vida	0.868 ± 0.021	0.865 ± 0.020	**8.91×10^{-11}**
Whole-image SSIM	Skyra Fit	0.825 ± 0.025	0.813 ± 0.024	**5.57×10^{-17}**
Whole-image MSE	Vida	0.0065 ± 0.0017	0.0062 ± 0.0017	**1.54×10^{-10}**
Whole-image MSE	Skyra Fit	0.0067 ± 0.0015	0.0070 ± 0.0016	**2.60×10^{-6}**
Lesion SSIM	Vida	0.374 ± 0.205	0.395 ± 0.215	0.457
Lesion SSIM	Skyra Fit	0.324 ± 0.209	0.325 ± 0.236	0.840
Lesion MSE	Vida	0.0858 ± 0.0878	0.0841 ± 0.1019	0.153
Lesion SSIM	Skyra Fit	0.1195 ± 0.1287	0.1371 ± 0.1315	0.122

Between-scanner comparison (Mann-Whitney U)

**Metric**	**Network**	**Comparison**	**p-value**
Whole-image SSIM	*vCE-clean*	Vida vs Skyra Fit	**3.98×10^{-25}**
Whole-image SSIM	*vCE-all*	Vida vs Skyra Fit	**4.11×10^{-30}**
Whole-image MSE	*vCE-clean*	Vida vs Skyra Fit	0.113
Whole-image MSE	*vCE-all*	Vida vs Skyra Fit	**1.59×10^{-5}**
Lesion SSIM	*vCE-clean*	Vida vs Skyra Fit	0.342
Lesion SSIM	*vCE-all*	Vida vs Skyra Fit	0.204
Lesion MSE	*vCE-clean*	Vida vs Skyra Fit	0.647
Lesion SSIM	*vCE-all*	Vida vs Skyra Fit	0.122
