# Supplementary material for: Influence of dominant sequence artifacts on generative AI for virtual contrast-enhancement in breast MRI
Source: BMC Med Imaging. 2026 Nov 3;26:578. doi: 10.1186/s12880-026-02996-8 (PMC13729665; doi:10.1186/s12880-026-02996-8)
Supplement: Supplementary file 5 [file 12880_2026_2996_MOESM5_ESM.pdf]

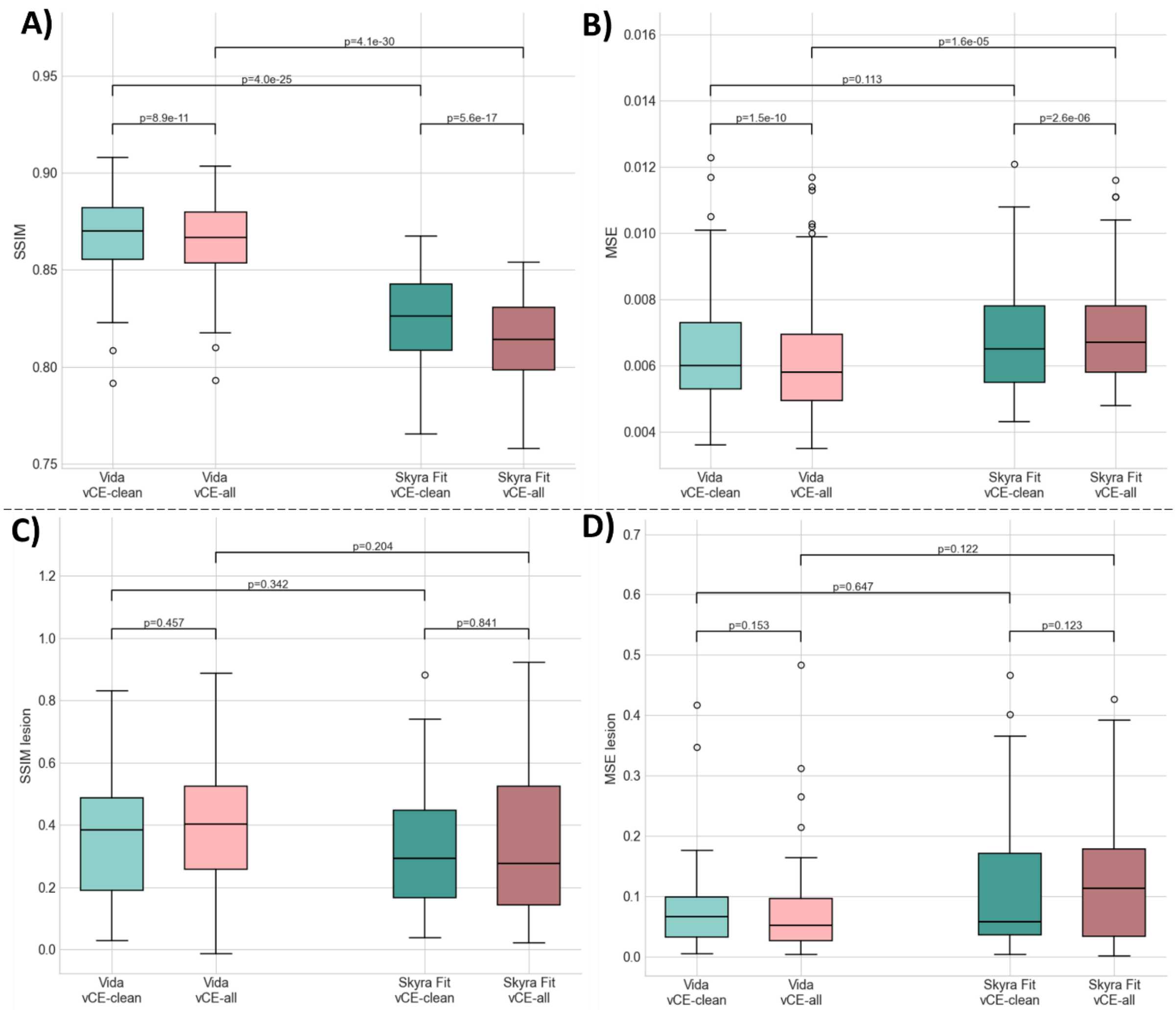

S4 Fig. Scanner-stratified quantitative performance of the virtual contrast-enhanced (vCE) models. Boxplots compare the performance of the vCE-clean and vCE-all models for examinations acquired on the MAGNETOM Vida and MAGNETOM Skyra Fit MRI systems. **A)** Whole-image structural similarity index (SSIM), **B)** whole-image mean squared error (MSE), **C)** lesion-based SSIM, and **D)** lesion-based MSE. Exact p-values for both within-scanner comparisons (vCE-clean vs. vCE-all) and between-scanner comparisons (MAGNETOM Vida vs. Skyra Fit) are indicated in each panel.
