# Supplementary material for: Influence of dominant sequence artifacts on generative AI for virtual contrast-enhancement in breast MRI
Source: BMC Med Imaging. 2026 Nov 3;26:578. doi: 10.1186/s12880-026-02996-8 (PMC13729665; doi:10.1186/s12880-026-02996-8)
Supplement: Supplementary file 6 [file 12880_2026_2996_MOESM6_ESM.pdf]

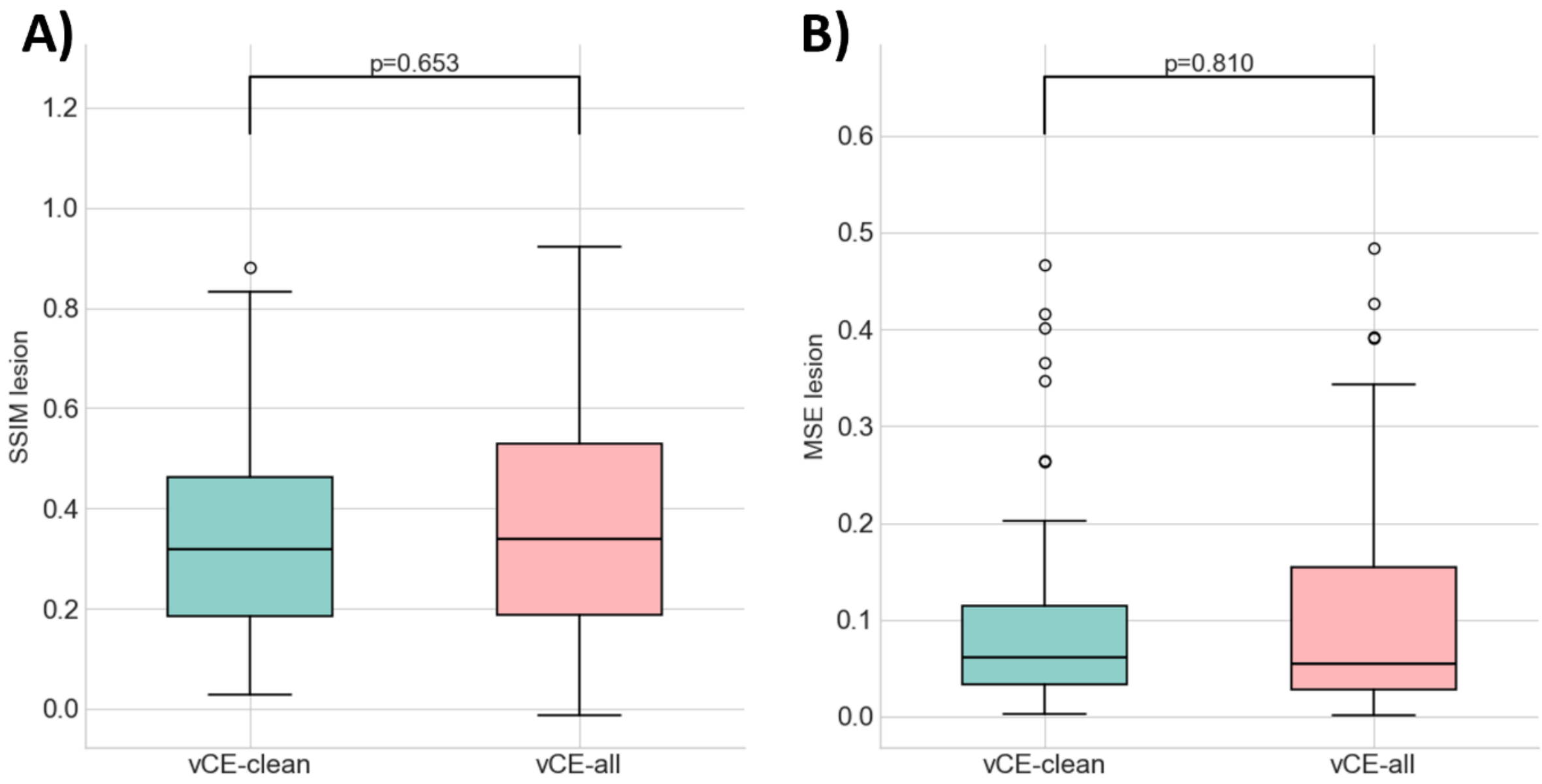

S2 Fig. Boxplots of Quantitative Evaluation Metrics Inside the Lesion Segmentations. The shown metrics are comparing the two virtual contrast-enhancement (vCE) approaches using “artifact-free” DWI data (*vCE-clean*) and the baseline approach using all available data (*vCE-all*). **A)** plots the SSIM of the lesion segmentation with no statistical significance. **B)** plots the MSE of the lesion segmentation with no statistical significance.
