# Supplementary material for: Influence of dominant sequence artifacts on generative AI for virtual contrast-enhancement in breast MRI
Source: BMC Med Imaging. 2026 Nov 3;26:578. doi: 10.1186/s12880-026-02996-8 (PMC13729665; doi:10.1186/s12880-026-02996-8)
Supplement: Supplementary file 7 [file 12880_2026_2996_MOESM7_ESM.pdf]

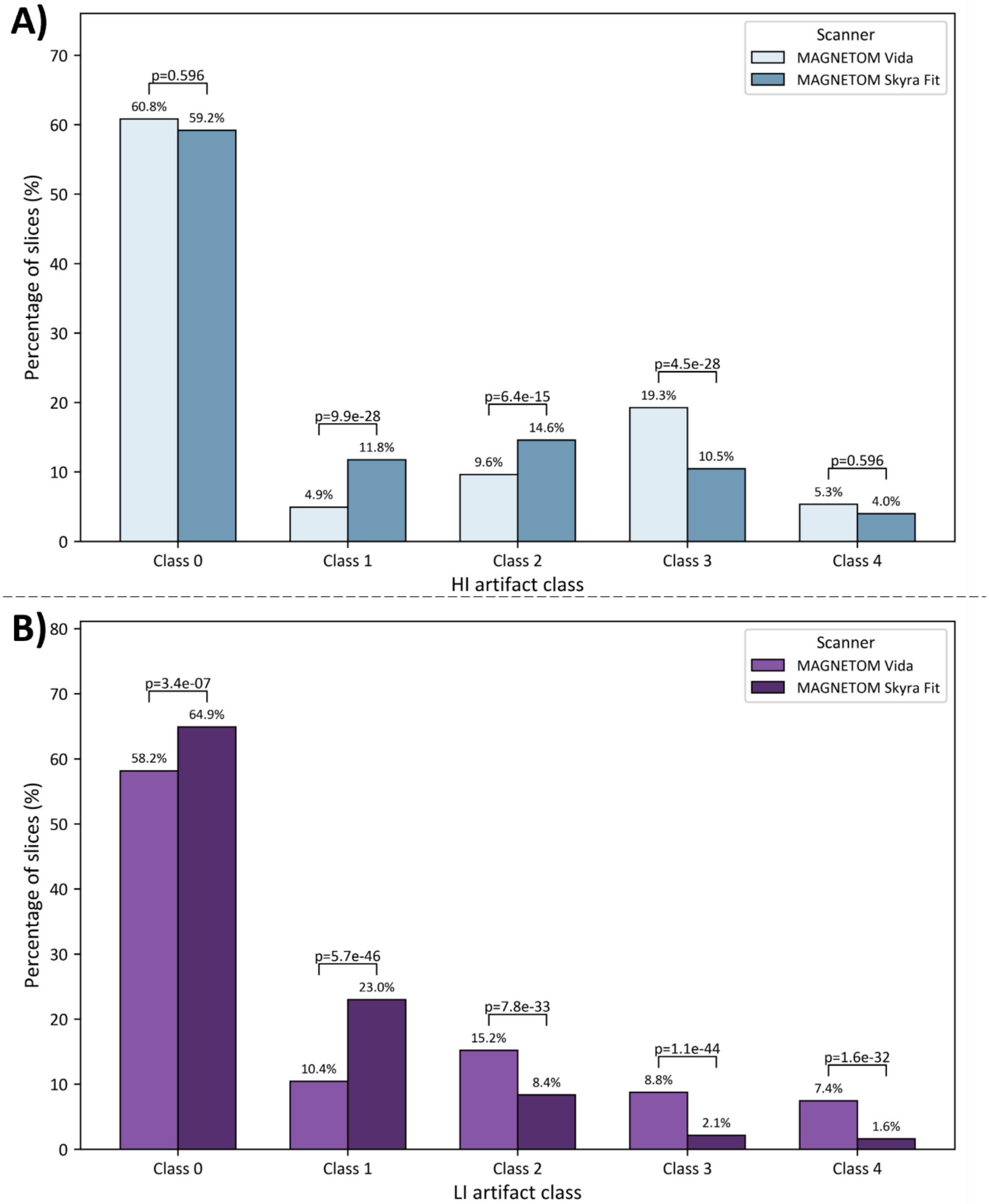

S3 Fig. Scanner-specific distribution of diffusion-weighted imaging (DWI) artifact severity. A) Distribution of high-intensity (HI) artifact classes and **B)** low-intensity (LI) artifact classes for examinations acquired on the MAGNETOM Vida and MAGNETOM Skyra Fit MRI systems. Bars represent the percentage of image slices assigned to each artifact class (0–4) by the pretrained artifact classification network. Statistical comparisons between scanners were performed on examination-level artifact percentages using Mann–Whitney U tests with Holm correction for multiple comparisons.
